# The impact of lifestyle Physical Activity Counselling in IN-PATients with major depressive disorders on physical activity, cardiorespiratory fitness, depression, and cardiovascular health risk markers: study protocol for a randomized controlled trial

**DOI:** 10.1186/s13063-019-3468-3

**Published:** 2019-06-20

**Authors:** Markus Gerber, Johannes Beck, Serge Brand, Robyn Cody, Lars Donath, Anne Eckert, Oliver Faude, Xenia Fischer, Martin Hatzinger, Edith Holsboer-Trachsler, Christian Imboden, Undine Lang, Sarah Mans, Thorsten Mikoteit, Anja Oswald, Uwe Pühse, Sofia Rey, Ann-Katrin Schreiner, Nina Schweinfurth, Ursula Spitzer, Lukas Zahner

**Affiliations:** 1 University of Basel, Basel, Switzerland; 2 Psychiatric Clinic Sonnenhalde, Riehen, Switzerland; 3 University of Basel, Adult Psychiatric Clinics (UPKE), Basel, Switzerland; 4 Kermanshah University of Medical Sciences (KUMS), Kermanshah, Iran; 5 German Sport University Cologne, Cologne, Germany; 6 Psychiatric Services Solothurn, Solothurn, Switzerland; 7 Private Clinic Wyss, Münchenbuchsee, Switzerland

**Keywords:** Acceptability, Biomarkers, Cardiorespiratory fitness, Cardiovascular risk markers, Counselling, Depression, Exercise, In-patients, Physical activity, Psychiatry, Serotonin transporter polymorphic promoter region

## Abstract

**Background:**

Major depressive disorder (MDD) is a widespread and burdensome psychiatric issue. Physical activity counselling may increase lifestyle physical activity and cardiorespiratory fitness in this specific and particularly vulnerable population, which often suffers from both mental and physical health problems. Therefore, this study will examine the impact of a lifestyle physical activity counselling intervention on physical activity, cardiorespiratory fitness, depression, and cardiovascular health risk markers among in-patients diagnosed with MDD compared to controls. Secondary purposes are to examine the acceptability and perceived usefulness of the intervention among these patients, to find out whether the effectiveness of the intervention is moderated by genetic factors, and to compare baseline values with an age- and gender-matched group of healthy controls.

**Methods:**

The study is designed as a multi-centric two-arm randomized clinical trial including an intervention group and a placebo control group, allocation concealment, single-blinding, and intention-to-treat analysis. Participants (*N* = 334) will be continuously recruited from four clinics specialized in the treatment of MDD. The intervention builds on a standardized, theory-based, low-cost lifestyle physical activity counselling programme, which was specifically designed for an in-patient rehabilitation setting. The placebo control condition consists of general instructions about health-enhancing physical activity. Data assessments will take place 2–3 weeks after admission to in-patient treatment (baseline), and 6 weeks (post) and 12 months (follow-up) after discharge from in-patient treatment. The primary outcome is objectively assessed physical activity at follow-up.

**Discussion:**

Because regular physical activity has proven to be an important predictor of long-term response and remission in patients with major depression, we believe that our planned study may lay important groundwork by showing how individually tailored lifestyle physical activity counselling can be integrated into given clinical structures. Improving physical activity may have important implications for tackling metabolic and cardiovascular disease and increasing mood and cognitive functioning in this at-risk population, hence limiting the future burden of multiple chronic conditions. Increased physical activity may also reduce the likelihood of future depressive episodes. By moving towards the primary prevention of chronic physical conditions, much can be done to enhance the quality and quantity of life of people with MDD.

**Trial registration:**

ISRCTN, ISRCTN10469580. Registered on 3 September 2018.

**Electronic supplementary material:**

The online version of this article (10.1186/s13063-019-3468-3) contains supplementary material, which is available to authorized users.

## Background

Major depressive disorder (MDD) is a psychiatric issue characterized by the loss of interests and pleasure in activities that were otherwise interesting and pleasant for the individual. Further, individuals with MDD report impaired sleep, suicidal behaviour, cognitive impairments, social withdrawal, diffuse and complex pain syndromes and issues, along with loss of sexual interests. As shown by the World Health Organization [1], MDD is widespread and its course is often chronic. Estimates of lifetime prevalence for MDD range between 10 and 20%, and the 1-year prevalence varies between 5 and 10%, affecting people of all ages, genders, and socio-economic status [2, 3]. Depression is often recurrent, and the risk of relapse is also high. Significant associations have been reported between MDD, morbidity, disability, mortality, and suffering for patients and their families [4]. The WHO Global Burden of Disease study suggests that mild to moderate depressive disorder is associated with the second greatest number of life years lost due to premature death or disability (DALY) [5] and will most likely be the leading cause in 2030 [6]. Moreover, MDD is the leading cause of years of life lived with disability (YLD) in men and women [7].

Close links have been found between MDD and poor quality of life, high medical expenditure, and increased use of healthcare services [8]. Moreover, a high comorbidity with other chronic medical conditions has been found in patients with MDD, all of which put a considerable burden on the healthcare system. For instance, people with depression are more than twice as likely to develop metabolic conditions (e.g. diabetes and metabolic syndrome) and cardiovascular diseases [9–13]. MDD is also linked with impaired cognitive functioning, leading to declines in information processing and reduced memory functioning [14].

As a possible moderator for both disease vulnerability and treatment response on the one hand, and occurrence of (somatic) comorbidities on the other, a dysfunction of serotonergic homeostasis has been proposed to play a crucial role [15–17]. Moreover, a polymorphic region in the promotor of the serotonin transporter (5-HTT) gene (5-HTTLPR), which is characterized by two alleles, a long (l) and a short (s) allele [18], has been suggested to be associated with the incidence of depression after exposure to stressful life events [17]. However, this association remains inconclusive, since some studies failed to replicate these findings [19, 20]. The inconsistencies might be due to unidentified environmental factors. In this regard, Rethorst et al. [21] recently found a significant interaction between the 5-HTTLPR genotype, physical activity, and depressive symptoms. Individuals with at least one s allele had significant higher levels of depressive symptoms at low-level physical activity compared to individuals with the ll genotype.

Further, people with both MDD and subthreshold depressive symptoms have an increased risk of premature mortality compared to the general population [22, 23]. There is a life expectancy gap of 10–15 years between psychiatric patients (including MDD) and people without psychiatric diagnosis. Importantly, around 80% of all preventable deaths are due to physical conditions, whereas suicide is only responsible for 14% [24]. Although these disparities have been recognized since the mid-1980s, this life expectancy gap has widened during the last 30 years [25]. Lawrence et al. [24] therefore concluded that “public efforts should be directed towards improving physical health to reduce mortality in people with mental illness, in addition to on-going efforts to prevent suicide” (p. 1).

### Standard treatment, remission rates, and complementary treatment options

Treatment options for MDD consist of pharmacological and non-pharmacological interventions [26], including psychotherapy, neuromodulation [27], physical activity [28], and nutritional supplements such as adjuvant omega-3-polyunsaturated fatty acids [29], or the combination of both pharmacological and non-pharmacological treatments [30–32]. However, the effectiveness of standard pharmacological treatment is limited [33], and the use of antidepressant medication is associated with side effects [34] and poor adherence [35]. It has been estimated that only about 30–50% of all patients show a response to a first antidepressant trial with single-action or dual-action antidepressant monotherapy [36, 37]. Remission is found in an even smaller portion of participants (15–40%) [37, 38]. In other words, more than 50% of all patients do not respond after first-line treatment. Current clinical practice employs switch, combination, and/or augmentation strategies after failure of first-line treatment. These augmentations include co-administration of antidepressants with alternative pharmacology, atypical neuroleptics, or mood stabilizers [8]. However, even these additional treatments often do not result in remission [39].

In light of the massive burden associated with MDD, the low rate of full recovery is problematic [37]. Researchers have therefore claimed that a greater variety of cost-effective, accessible, and alternative/complementary treatments are needed [40, 41]. A further justification for this claim comes from the studies which found relatively low compliance with antidepressant medication, indicating that 20–60% of primary care patients stop taking their medication within the first 3 weeks after drug prescription [42]. One important reason is that the use of psycho-pharmaceuticals may have negative side effects, such as clinically significant weight gain and, above all, SSRI-related sexual dysfunction [43, 44]. Specifically, weight gain is of particular relevance, as it is associated with reduced quality of life, social stigma, greater morbidity (e.g. cardiovascular disease, diabetes mellitus, osteoarthritis), and mortality [45].

As a consequence, non-pharmacological and complementary strategies have been envisaged to improve the prognosis of MDD [26], including exercise therapy [28]. The use of alternative and complementary treatments is popular and widespread in western societies. For instance, in a US-based sample of severely depressed participants, more than half indicated having used such options during the past year [46], most likely because these alternatives are consistent with their own values, beliefs, and philosophical orientations towards health and life [47]. Part of such orientation is physical activity, and therefore, according to Dunn et al. [48], exercise seems a viable treatment option because it “can be recommended for most individuals, and does not carry a negative social stigma” (p. 1).

### Exercise as an alternative or complementary therapy in the treatment of MDD

In several countries, health foundations have encouraged general practitioners to prescribe exercise as a front-line strategy in the treatment of MDD [40, 49, 50]. Research also shows that regular exercise and physical activity have the potential to reduce the risk of developing depression [51, 52]. Meanwhile, several meta-analyses have examined the effects of exercise on depressive symptoms in randomized controlled trials (RCTs) among adult populations. The first meta-analysis was published in 2005 by Lawlor and Hopker [49]. Based on 14 studies, their findings showed that exercise treatment was associated with an effect size of *d* = − 1.10, pointing towards a significant reduction in depressive symptoms compared to no treatment. However, a higher effect size was found in studies with shorter follow-up periods. Using a more extensive search procedure, Rethorst et al. [40] identified 58 RCTs and found an overall effect size of *d* = − 0.80, highlighting that exercise treatment results in significantly decreased depression scores compared to controls.

In a recent Cochrane review, Cooney et al. [53] found similar effects (*d* = − 0.62) when comparing exercise to standard treatment, no treatment, or placebo control. However, after excluding trials which did not fulfil the adequate quality standards (such as allocation concealment, blinded outcome assessment, intention-to-treat analysis), the effect size was no longer significant (*d* = − 0.18). Nevertheless, those eight studies which presented long-term follow-up data resulted in a moderate effect (*d* = − 0.33) in favour of exercise treatment. Moreover, Schuch et al. [54] pointed out that publication bias may lead to an underestimation of the standardized mean difference reported in RCTs, and that programmes are particularly effective if they promote moderate intensity physical activity, if they have an emphasis on aerobic exercise activities, and if the interventions are provided and supervised by exercise professionals. Kvam et al. [55] summarized in their meta-analysis that physical activity interventions showed large effects sizes when compared to no interventions (*g* = 1.24), and moderate effect sizes when compared to control conditions (*g* = 0.68) and to usual care (*g* = 0.48). However, when compared to psychological interventions (*g* = 0.22) or antidepressant medication (*g* = 0.08), effect sizes were small. However, again when combined with antidepressant medication, effect sizes were moderate (*g* = 0.50). Further, and most importantly, Kvam et al. [55] showed that the effect of physical activity had a moderate to large significant effect on depression compared to control conditions (*g* = − 0.68), but that the effect was small and not significant at follow-up (*g* = − 0.22). The latter result clearly indicates the need for and necessity to build up a well-designed intervention to thoroughly monitor the transition from guided and supervised interventions during stays in hospital and the patients’ conditions and settings after discharge, when they should monitor the physical activity themselves. Evidence also supports that individual tailoring is recommendable, as both aerobic and anaerobic exercise activities have a similar potential to reduce depression [56, 57] and to foster exercise motivation [58].

Blumenthal et al. [4, 31] compared whether 4-month aerobic exercise programmes are more effective than pharmacotherapy in two separate studies with 156 older patients and 202 adults with MDD. Taken together, these studies reveal that exercise and antidepressant medication lead to similar reductions in depressive symptoms, and that both treatments are associated with higher remission rates compared to a placebo control condition [4]. Beyond these short-term outcomes, Hoffman et al. [59] examined the long-term effects of the different treatments. While neither group assignment (exercise vs pharmacotherapy) nor antidepressant medication usage during the follow-up period were related to response or remission at 12-month follow-up, regular exercise during the follow-up period proved to be the only significant predictor. In other words, patients who regularly engaged in exercise activities after the initial treatment were less likely to have MDD at follow-up. These results corroborate previous research with older adults, showing that regular post-treatment exercise leads to a considerably lower relapse rate 6 months after the end of the treatment [60].

While these results suggest that exercise therapy can be equally effective in reducing depressive symptoms as antidepressants in the short run and that prolonged regular exercise might prevent relapses after the end of treatment, it is evident that the positive effects of exercise therapy may dissipate if the intervention is discontinued and if patients are not able to maintain a physically active lifestyle.

### Effects of exercise training on cardiorespiratory fitness and cardiovascular health

A physically active lifestyle is associated with increased cardiorespiratory fitness (CRF), and high levels of CRF are associated with a reduced risk of cardiovascular morbidity and other chronic conditions such as obesity, diabetes, cognitive decline, or specific forms of cancer [61]. Moreover, high CRF is associated with reduced risks of premature mortality, even after controlling for the influence of hereditary factors [62, 63].

Nevertheless, several studies show that people with depression have lower physical activity [64, 65] and CRF levels [66, 67]. Thus, it has been hypothesized that low physical activity and CRF constitute a possible link between depression and comorbid somatic disorders [68, 69]. Importantly, a meta-analytic study revealed that exercise interventions significantly contribute to increased CRF in patients with MDD [70]. Based on seven RCTs, an overall effect size of 0.64 was found, which corresponds to a mean increase of 3.05 ml/kg/min of oxygen uptake. As shown in population studies, improvements of 3.5 ml/kg/min in VO_2_max are associated with a 13 and 15% decrease in cardiovascular disease and all-cause mortality [63].

### Summary of main findings and special challenges in patients with major depressive disorder

Strong empirical evidence exists showing that exercise plays a beneficial role in the treatment of MDD. Exercise, antidepressants, and psychotherapy have comparable effects among patients with MDD [71], although, above all, the combination of both physical activity and standard medication treatment appears to be particularly promising [55]. Further, exercise might even be successful in reducing treatment-resistant depression [28]. Given these observations, it seems recommendable to use exercise more systematically as an add-on to standard care during in-patient treatment [50, 55, 72]. Moreover, promoting lifestyle physical activity seems to have the potential to prevent relapses after the end of hospitalization [59, 73].

Initiating and maintaining regular exercise among patients with MDD is a major challenge, especially because depressive symptoms interfere with their capacity to self-regulate health-related behaviours [33, 68]. MDD is often cyclical, including recurrent depressive episodes, which might lead to an interruption of exercise regimes. Moreover, depression can be linked with motivational and volitional deficits in all areas of daily life due to hopelessness, pessimism, loss of interest and enjoyment in ordinary things, persistent low mood, low self-efficacy, limited capacity to plan due to impaired executive function, and a tendency to postpone tasks [33, 68]. In line with this, studies show that depression is associated with limited exercise self-efficacy, stronger negative outcome expectations, reduced intentions to exercise, poor maintenance self-efficacy, and increased perception of situational barriers [68, 74]. At the same time, higher body mass index (BMI), and the presence of somatic comorbidities are particularly important barriers for people with depression to engage in regular physical activity [75]. In other words, engagement with and adherence to recommended physical activity levels remain a major challenge. Providing MDD patients with professional support to identify and achieve their physical activity and exercise goals may enable them to overcome psychological barriers, and maintain motivation towards regular physical activity. Thus, Gerber et al. [76, 77] suggested that looking into lifestyle physical activity counselling should become a top priority to improve patients’ behavioural skills such as action planning, coping with exercise-related barriers, and social support. Such behavioural skills are key to maintaining a more physically active lifestyle and to achieve long-term positive outcomes in mental health.

### Physical activity counselling and behavioural skill training to foster regular exercise participation

Several theories have been developed to facilitate change in health behaviour [78]. Two recent models are the Health Action Process Approach model [79] and the Motivation and Volition-process model [80]. The common ground of these models is that they focus on both motivational (how people form an intention) and post-intentional (volitional) processes, including action planning, identification of personal and environmental barriers, coping planning, and relapse management. Both models have resulted in theory-based intervention programmes that provide support for long-term behaviour change in overweight people and patients suffering from somatic conditions [81–84]. As an example, Fuchs et al. [81] showed that amongst 220 inactive in-patients of an orthopaedic rehabilitation clinic, motivational and volitional counselling resulted in a moderate-to-large short-term increase (*d* = 0.72) in physical activity in the intervention group compared to the control group. Although the group difference diminished until 12-month follow-up, the intervention group still reported increased physical activity levels. Nevertheless, while these findings are promising, research about the potential of such programmes in patients with MDD is scarce.

A recent study showed that lifestyle counselling (including diet, exercise, sunlight exposure, and sleep) can be an effective add-on to antidepressant treatment. While remission reached 60% in the combined treatment group, this rate was considerably lower in the control group (10%) [85]. So far, only one RCT exists in which the effects of individually tailored physical activity counselling through professionally trained physical activity facilitators have been tested in patients with MDD [86–88]. While the findings showed that the intervention group had significantly increased physical activity levels at follow-up, this study was carried out in an out-patient setting, did not focus on patients with low physical activity levels, and did not assess physical activity objectively.

## Purpose of the study

Given this background, the main purpose of this study is to implement and evaluate a randomized controlled trial to examine the effectiveness of an individually tailored lifestyle physical activity counselling intervention on objectively assessed physical activity among in-patients diagnosed with MDD compared to (placebo) controls. Patients will receive exercise prescriptions tailored towards their current physical activity level, activity type, and intensity preferences in order to change their exercise and physical activity habits beyond their stay at the clinic. To ensure a high adherence among patients assigned to the intervention group across the entire study period, new technologies such as an app-based coaching platform and remote physical activity counselling (telephone coaching and message prompts) will be used [89].

Secondary purposes are as follows:

- to examine how the intervention impacts on the secondary outcomes (self-reported physical activity, cardiorespiratory fitness, autonomic function, cognitive and social determinants of exercise, depression severity, self-perceived physical and psychological health, insomnia symptoms, cognitive function, cardiovascular risk profile, and biomarkers of MDD);
- to find out whether the serotonin transporter (5-HTT) polymorphic promoter region (5-HTTLPR) as a genetic factor moderates the effects of the intervention;
- to gain insights into the acceptability and perceived usefulness of the intervention among patients; and
- to compare baseline values with an age- and gender-matched group of healthy controls.

## Methods/design

### Study design

**Fig. 1:**
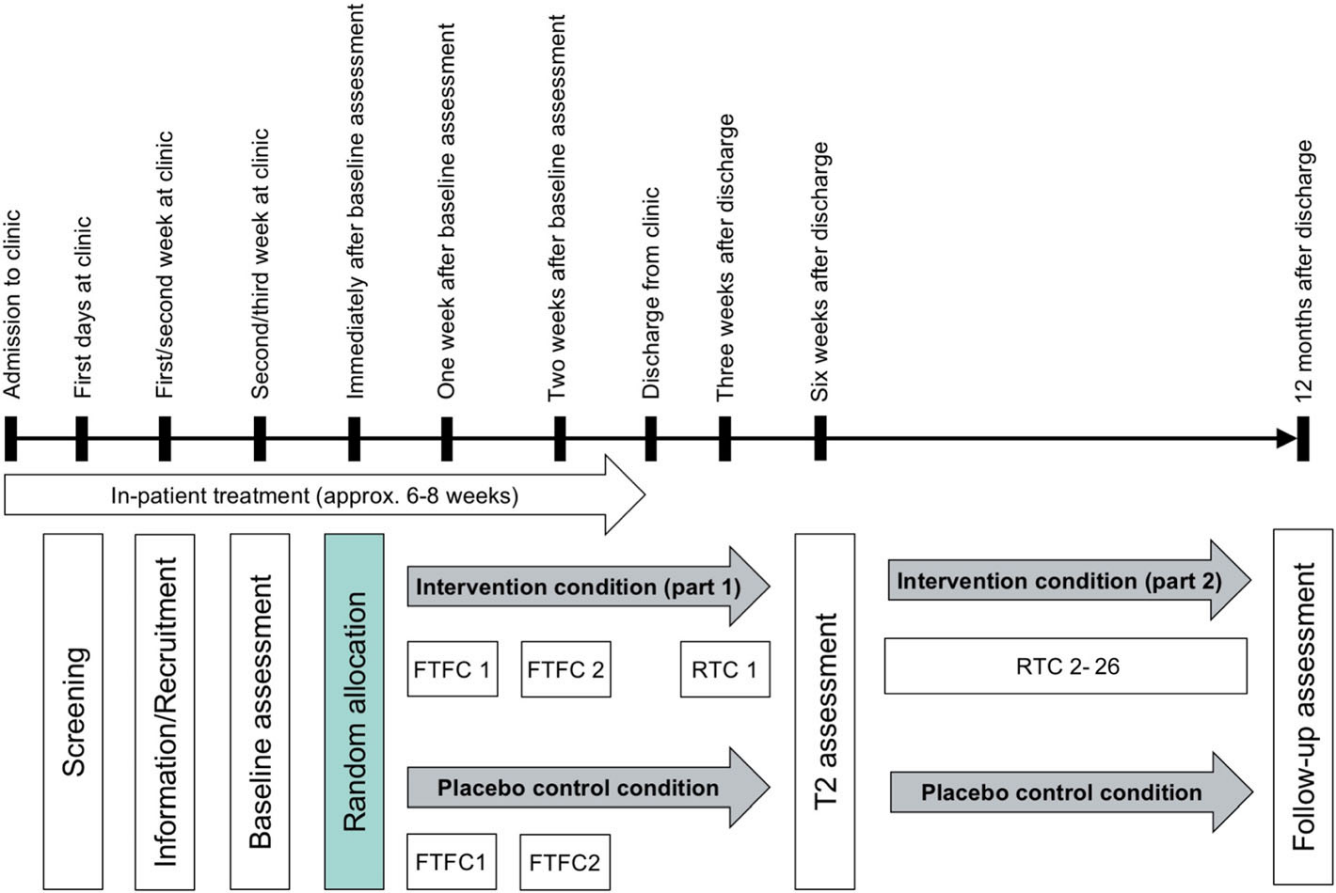
Overview of the planned randomized controlled trial study design. FTFC face-to-face counselling with coach (behaviour skill training for intervention group; written information and video clip about health-enhancing physical activity for placebo control group), RTC remote telephone counselling, T2 6 weeks after discharge

**Fig. 2:**
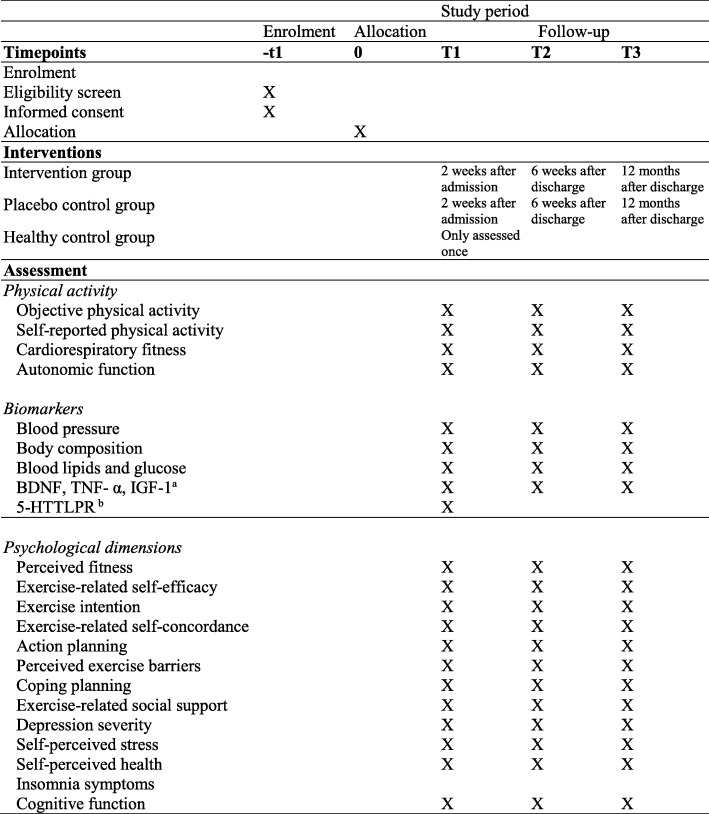
SPIRIT figure providing an overview of time points, interventions, and assessments of the PACINPAT randomised controlled trial. BDNF brain-derived neurotrophic factor, 5-HTTLPR serotonin transporter polymorphic promoter region, IGF-1 insulin-like growth factor 1, PACINPAT Physical Activity Counselling in IN-PATients, SPIRIT Standard Protocol Items: Recommendations for Interventional Trials, TNF-α tumor necrosis factor alpha

The study is designed as a multi-centric, two-arm RCT including an intervention group (IG) and a placebo control group (PCG), allocation concealment, single-blinding, and intention-to-treat analysis. The study is a cooperation between four Swiss psychiatric clinics (two public, two private) and the Department of Sport, Exercise and Health of the University of Basel. Figure 1 provides an overview of the planned study design. The 25-item CONSORT checklist was used when designing the study. Figure 2 represents the SPIRIT figure, providing an overview of foreseen time points, interventions, and assessments. The description of the study protocol contains all elements listed in the SPIRIT checklist (see Additional file 1).

Patients will be assigned randomly (stratified by age, gender, and clinic) to an extended personalized physical activity and exercise counselling programme (IG) or to general instructions about health-enhancing physical activity (PCG). To ensure allocation concealment, allocation to groups will be done by a computer-generated code after the baseline assessment has taken place. A researcher (OF) will generate an allocation sequence and assign participants to either the intervention or placebo control group. Further researchers engaged in field work (RC and SR) will recruit and enrol participants. Clinicians, researchers, and physical activity facilitators will not be blinded to treatment allocation because of the nature of the study.

To minimize subjective bias, patients do not know whether they have been allocated to the intervention or placebo control group. Before providing informed consent, patients will be informed that the goal of the study is to test different methods to promote a more physically active lifestyle among patients with MDD who do not meet levels of physical activity recommended by the American College of Sports Medicine [90, 91]. Outcomes will be assessed by researchers who do not know whether a patient belongs to the IG or the PCG.

### Participants

#### Recruitment and power calculations

Clinical in-patients aged 18–65 years with a current diagnosis of major depression (ICD-10) will be continuously recruited from the Psychiatric Services Solothurn, Psychiatric Clinics of the University of Basel, Psychiatric Clinic Wyss, Münchenbuchsee, and the Psychiatric Clinic Sonnenhalde, Riehen. In previous studies with non-depressed individuals, individually tailored physical activity promotion was associated with a moderate effect (*d* = 0.50) on the primary outcome (physical activity) [80, 81]. So far, only one study exists examining physical activity counselling among out-patients with depression, yielding an adjusted odds ratios of 2.27 across the 4-month, 8-month, and 12-month follow-up (corresponding *d* = 0.45). Conversely, a relatively small effect size (*d* = 0.28) was observed in a Cochrane review of intervention studies promoting physical activity among generally healthy people [92]. Similarly, remote interventions such as telephone-based, app-based, and Internet-based coaching versus a control group were associated with rather small effect sizes for change in physical activity (*d* = 0.20) [93]. Given these observations, we assume a small-to-moderate effect of our intervention on the primary outcome (*d* = 0.30). Accordingly, the optimal sample size to detect a significant effect at follow-up is 278 participants (two independent groups, one-tailed, α-error probability = 0.05, power = 0.80). With 20% expected dropouts from baseline to follow-up [94], the targeted sample size is *N* = 334 (IG, *n* = 167: PCG, *n* = 167). This means that every centre will recruit approximately 40 participants per year (*n* = 20 in the IG, *n* = 20 in the PCG). Dependent on the size of the clinics, this corresponds to a recruitment rate of 20–25% of the patients. Enrolment will be systematically monitored by the sponsor-investigator in order to recruit a sufficient number of participants within the planned timeframe.

#### Screening

A structured clinical interview will be conducted by a psychiatrist to ensure that all participants fulfil the ICD-10 diagnosis for a single episode (F32), recurrent MDD (F33), or bipolar disorder type II (F31-II). The screening also includes assessment of the duration of the current depressive episode, the number of previous depressive episodes, psychiatric and somatic comorbidities, and family history of psychiatric and somatic conditions. To assess symptom severity, a trained staff member will apply the 17-item Hamilton Depression Rating Scale (HAMD17) [95] as part of a clinical interview [96], while patients’ self-ratings are based on the 21-item Beck Depression Inventory (BDI) [97, 98], along with the short version of the International Physical Activity Questionnaire (IPAQ) [99] to assess self-reported physical activity (referring to the last week before entering the clinic).

#### Criteria for inclusion/exclusion

Inclusion criteria are: women and men; 18–65 years of age; presence of MDD according to ICD-10 diagnostic criteria (F31 type II, F32, F33); BDI ≥ 17 (at least borderline clinical depression); currently not meeting the ACSM physical activity recommendations (IPAQ < 150 min/week of moderate-to-vigorous physical activity); written informed consent; and ability to speak and read German.

Exclusion criteria are: presence of history of bipolar disorder type I (F31 type I), history of schizophrenia, or schizoaffective disorder; current active alcohol or drug abuse or dependence; any significant medical condition that contraindicates safe participation in physical activity; active suicidal intent; evidence of significant cardiovascular, neuromuscular, or endocrine disorders limiting regular physical activity as per ACSM absolute contraindications to exercise (including a recent significant change in the resting ECG suggesting significant ischaemia, recent myocardial infarction or other acute cardiac event, unstable angina, uncontrolled cardiac dysrhythmias causing symptoms or haemodynamic compromise, symptomatic severe aortic stenosis, uncontrolled symptomatic heart failure, acute pulmonary embolus or pulmonary infarction, acute myocarditis or pericarditis, and suspected or known dissecting aneurysms) or medical contraindications to physical activity indicated by the Physical Activity Readiness Questionnaire (PAR-Q) [100]; and inability to speak and read German.

#### Healthy controls

To compare baseline differences in the primary and secondary outcome(s) between patients and healthy controls, an age- and gender-matched sample of 167 participants will be recruited through advertisements in newspapers and through word-of-mouth recommendation. Inclusion criteria for healthy controls are: women and men; 18–65 years of age; HAMD17 ≤ 7; BDI ≤ 13; currently not meeting the ACSM physical activity recommendations (IPAQ < 150 min/week of moderate-to-vigorous physical activity); written informed consent; and ability to speak and read German.

### Procedures

#### Data assessment

Screening will take place in the first week after admission to in-patient treatment. Identical data assessment will take place 2–3 weeks after admission to in-patient treatment (baseline), and 6 weeks (post) and 12 months (follow-up) after discharge from in-patient treatment. The primary outcome is objectively assessed physical activity (Actigraph wGT3x-BT monitor, 7 days). Secondary outcomes are self-reported physical activity, cardiorespiratory fitness, autonomic function cognitive and social determinants of exercise, depression severity, self-perceived physical and psychological health, insomnia symptoms, cognitive function, cardiovascular health risk markers, and biomarkers of MDD. Moreover, the serotonin transporter (5-HTT) polymorphic promoter region (5-HTTLPR) is assessed as a potential moderator of intervention effects [21]. More information regarding the various instruments is presented in the following.

### Intervention and placebo control condition

Patients assigned to the IG or the PCG receive treatment as usual from their psychiatrists according to the Swiss treatment guidelines for major depression [101]. Moreover, patients from both groups continue the therapy provided by their practitioner and therapist for their depression (i.e. psychotherapy, use of antidepressants).

#### Intervention programme: individually tailored physical activity promotion

Patients assigned to the IG will receive an individually tailored physical activity counselling to provide support and encouragement to increase physical activity. Two face-to-face meetings will take place during the clinical treatment which lasts between 6 and 8 weeks on average. The meetings will take place approximately in weeks 4 and 6 of in-patient treatment. The same physical activity facilitator will develop a physical activity plan with the participant, tailored to the individual’s current activity levels, activity type, and intensity preferences, and will promote behavioural change techniques which have been proven effective in previous studies [102–107]. Additionally, starting 1 week after discharge from in-patient treatment, patients of the IG will receive bi-weekly telephone counselling and SMS prompts until 12 months after discharge. Telephone counselling and push notifications will help to ensure adherence of patients to their physical activity and exercise plans/prescription, or facilitate the adaptation of these plans. Furthermore, a smartphone app will be developed which can be used to support patients in their planning and self-monitoring of physical activity and exercise behaviour.

Exercise prescriptions follow the standards of the American College of Sports Medicine [90, 91], stating that individuals should engage in at least 30 min of moderate-intensity physical activity on 5 days per week, or 20 min of vigorous-intensity aerobic exercise on 3 days each week. A variety of activities such as aerobic exercise, resistance exercise, multi-modal group-based exercise, daily physical activity (e.g. walk or take bike for grocery shopping), and so forth, will be advised.

The intervention builds on a standardized, theory-based, short, low-cost lifestyle physical activity counselling programme, which was specifically designed for an in-patient rehabilitation setting (MoVo-Lisa = Motivation, Volition and Lifestyle-integrated Sport Activity). It consists of two individual (face-to-face) counselling sessions and an extended counselling session via telephone after discharge from the clinic. The first session (60–75 min) will take place 1 week after the baseline data assessment. The second session (45–60 min) will take place 1 week before the end of in-patient treatment. The third session (75–90 min) will take place 1–2 weeks after discharge from in-patient treatment via telephone to give patients the possibility to test their physical activity plans in their familiar environment, and to allow the identification of real-life physical activity obstacles.

All sessions aim at promoting motivational and volitional strategies to foster long-term behavioural change. Following Michie et al.’s [102] behaviour change taxonomy, motivational strategies include: clarification of personal health objectives (*goal setting [outcome]*, V1: 1.3); contemplation of different actions to achieve the health objectives (*goal setting [behaviour]*, V1: 1.1); reflection of pros and cons associated with the new behaviour (*pros and cons*, V1: 9.2); checking self-concordance of these goals (*review behaviour/outcome goals*, V1: 1.5/1.7); and reflection of outcome experiences (*monitoring of emotional consequences*, V1: 5.4). Volitional strategies include: generation of implementation intentions (*action planning*, V1:1.4); anticipating personal barriers (*problem-solving*, V1:1.2); developing counter strategies (*restructuring of the physical/social environment*, V1:12.1/12.2); contracting (*behavioural contract*, V1:1.8); mobilization of social support (*social support*, V1:3); and self-monitoring the new behaviour (*self-monitoring of the behaviour and outcomes of the behaviour*, V1:2.3/2.4). The intervention will be designed to match the specific stage of depression. Facilitators will focus on pessimistic beliefs such as negative outcome expectations and self-efficacy, as well as planning deficits linked with depression [108]. Recently, reviewers identified losing weight (83% of patients), improving mood (81%), and reducing stress (78%) as the most relevant motives for exercise in patients with severe mental illnesses, whereas low mood and stress (61%) and lack of social support (50%) turned out to be the most relevant exercise barriers [69]. Physical activity facilitators will place a special emphasis on these motives and barriers when developing physical activity promotion strategies.

#### Administration of experimental intervention

The aim is to have at least two face-to-face individual counselling sessions, have 26 follow-up telephone contacts (on a bi-weekly basis), and send 52 message prompts by the 12-month time point. Telephone contacts will be used to discuss problem-solving around barriers, reinforce progress towards behaviour change, and adapt initial goals and plans. Moreover, follow-up contacts serve to strengthen relapse strategies so that patients understand that relapses are common during behaviour change, and that dysfunctional cognitions, emotion, and behaviours can jeopardize maintenance of a physically active lifestyle [33].

#### Smartphone application

A smartphone app will support patients in their planning and self-monitoring of physical activity and exercise behaviour. This newly developed app designed for online coaching will serve as a tool for communication, exchange of weekly activity plans, and documentation of physical activity. Via the app, the physical activity facilitator can assist the participant in developing physical activity plans and can access the self-monitoring data. Both participants and physical activity facilitators can access and modify the physical activity plans online.

Adherence to the intervention protocol will be monitored by the physical activity facilitators. A thorough documentation of the physical activity facilitator’s work with the participant will ensure that the adherence to the protocol will be visible. Bi-weekly meetings with the physical activity facilitators and the study team will allow for continued training and minimize differences in the delivery of the intervention between facilitators and within facilitators over time.

#### Placebo control condition

One week after the baseline assessment, in addition to treatment as usual, patients assigned to the PCG will receive written information about health-enhancing physical activity based on the “Core document for Switzerland” published by the Federal Office of Sport (BASPO) in collaboration with other institutions. This document summarizes the current knowledge in relation to health-enhancing physical activity. The patients will obtain information about the following topics: why is physical activity healthy; what are the minimal physical activity recommendations; how physically active are people in Switzerland; and what are the costs of physical inactivity?

One week before the end of the in-patient treatment, patients will meet the physical activity facilitator. The facilitator will summarize the key contents of the “core document” by showing a short animation film (https://www.youtube.com/watch?v=zNZenOnGI0U) and discuss questions with the patient. The control condition is not intended as a bona fide intervention [109, 110] and an active therapy, but to control for placebo effects of the intervention condition.

#### Selection, education, and training of the physical activity facilitator

Physical activity counselling will be done by personnel with a background in exercise science and/or psychology, because a majority of patients want professional support when trying to engage in more physical activity [69]. Physical activity facilitators will be trained in behavioural change techniques and coaching through the supervisors. The facilitators will be regularly supervised by the investigators. Knowledge and coaching style of each facilitator will be tested prior to the first coaching session.

#### Participation in physical activity and exercise programmes during in-patient treatment

The physical activity facilitator will inform all participants about the existing exercise activities offered at the clinic. Both groups (IG and PCG) have the possibility to voluntarily participate in these activities.

### Ethical considerations

The proposed research will be carried out in accordance with the ethical principles laid down in the Declaration of Helsinki (1964). Central ethical approval has been confirmed from the “Ethikkommission Nordwest- und Zentralschweiz” (ref. approval no. 2018-00976). Moreover, local ethical approval has been obtained for all study sites. Thus, local ethical approval has been obtained from the “Ethikkommission Nordwest- und Zentralschweiz” for the clinics located in Basel, Riehen, and Solothurn, and from the “Ethikkommission Bern” for the clinic located in Münchenbuchsee. The intervention study was registered in the WHO trial register (trial number ISRCTN10469580). All participants will be informed about the general goals of the study. Informed written consent is required before study entry. Participants are informed that participation in the study is voluntary and that they can withdraw or discontinue at any time without further obligation or potential disadvantages. The participants and outcome assessors are blinded. Medication intake will be recorded as a part of the baseline, post intervention, and follow-up assessments.

After completion of the data assessment, all personal data of the patients will be encoded (each patient will receive a project ID number), so that it will no longer be possible to identify the patients. Data will be entered into an SPSS file. The collected data will be saved digitally. Backup files will be stored regularly on the external cloud *Switchdrive@Universität Basel*. The coding list will be stored in a safe place. Data will only be used for scientific purposes, and will be discarded after completion of the laboratory analyses. Paper records of the study will only be accessible to the main investigators, and will be kept in locked cupboards. After 10 years, all records will be destroyed.

### Measures

The primary outcome, secondary outcomes, moderators, and covariates will be assessed identically at baseline, post intervention, and follow-up by the same examiner at the clinic. As an incentive, healthy controls will receive 50 CHF for their participation in the study. Patients will receive 20 CHF if they participate in the post-intervention and follow-up assessments (as a compensation for their travel expenses). After each data assessment, healthy controls and patients will receive a health profile with information regarding their physical activity and fitness level, blood pressure, blood glucose, blood lipid profile, and body composition. Participants will be instructed to abstain from food and liquids (including coffee and tea) from 22:00 the night before the evaluation for the accurate measurement of blood glucose, blood lipids, and autonomic function. Assessments will last approximately 2 h. The following procedures will be applied by well-trained staff adhering to standardized, quality-controlled protocols.

### Primary outcome

#### Objective physical activity

Objective physical activity will be assessed with an accelerometer (wGT3x-BT; Actigraph, Shalimar, FL, USA) worn around the hip. The devices will be worn during daytime for 7 consecutive days to assess a full weekly period. The sampling epoch will be set at 10 s [111]. Time per day spent in moderate physical activity (MPA; 1952–5723 counts per minute, > 3 MET) and vigorous physical activity (VPA; > 5274 counts per minute, > 6 MET) is determined based on the raw accelerometer counts and the ActiLife® computer software, with cut-off values derived from Freedson et al. [112]. Participants will fill in a non-wear time sheet (e.g. to assess physical activities during which it was not possible to wear the monitor, such as swimming). Physical activities listed on the non-wear time sheet will be included as moderate-to-vigorous physical activities, based on the intensity levels defined in the Physical Activity Compendium [113, 114]. To be included in the data analyses, participants will need at least 5 valid days, including ≥4 valid weekdays and ≥ 1 valid weekend day [115]. Following Herrmann et al. [116], only days with at least 10 h of wear time are considered a valid measure of daily physical activity. The validity of the Actigraph accelerometer device has been documented previously [117]. To the best of our knowledge, no minimal clinically important change (MCID) scores are currently available for physical activity in patients with MDD. The ACSM recommends that individuals should accumulate at least 150 min of moderate-to-vigorous physical activity per week [91]. This is in line with a study by Hoffman et al. [59] showing that in patients with MDD there is a linear increase associated with self-reported exercise regarding the probability of at least partial remission until 150 min of exercise per week, whereas the response curve flattens off beyond this threshold.

### Secondary outcomes

#### Self-reported physical activity

Self-reported physical activity will be assessed with a newly developed questionnaire (Simple Physical Activity Questionnaire (SIMPAQ)) specifically developed for the use with psychiatric patients [118]. Validation of this instrument with psychiatric patients is currently underway (see www.simpaq.org). Data for healthy people show that the instrument is reasonably associated with objectively assessed physical activity [119]. The SIMPAQ uses an interview format (5–10 min in duration) to assess time in bed, structured exercise participation, and incidental or non-structured physical activity. Data from our research group assessed in university students show that moderate-to-vigorous physical activity estimated with the SIMPAQ correlates moderately to highly with accelerometer data (*r* = 0.30–0.70) [119]. The same public health recommendations apply as for objectively measured physical activity [91].

#### Cardiorespiratory fitness

The Åstrand and Rodahl indirect test of maximal oxygen uptake (VO_2_max) will be used to assess cardiorespiratory fitness [120]. The test will be performed on a bicycle ergometer (Bike Forma; Technogym) at the same time of the day (starting between 08:00 and 10:00). This test has been validated for the purposes of measuring submaximal fitness [120]. The pedalling frequency during the Åstrand test will be set at 50 rpm, and the workload adjusted so that the heart rate is kept between 130 and 160 beats per minute (bpm) in participants younger than 40 years old and between 120 and 150 bpm in participants older than 40 years old. To ensure that participants maintain their exercise intensity level at 13 or 14 (slightly strenuous), we will employ the Borg Rating of Perceived Exertion scale [121]. When the heart rate remains stable after 5 or 6 min, a steady state is reached. Peak oxygen uptake (l/min) will be estimated based on mean steady state, sex, and power output, using a nomogram [120], and including a correction factor for age. After correction of body weight, oxygen uptake will be expressed as peak VO_2_max (ml/kg/min). Gender and age-adjusted cut-off values will be used to categorize participants into groups with low, moderate, and high CRF [66]. The reliability and validity of the Åstrand nomogram and linear extrapolation for deriving VO_2_max has been documented in a previous study [122].

#### Autonomic function

Using heart rate monitors (V800; Polar Electro, Finland), following a 5-min period of rest, R–R intervals will be recorded over 5 min; based on their variation, the heart rate variability (HRV) is calculated. This non-invasive method allows for the accurate evaluation of autonomic nervous system activity [123]. Recorded R–R intervals will be processed and analysed with Kubios HRV [124]. Evidence for the validity of the Polar V800 monitor for the assessment of HRV at rest has been documented in a prior study [125]. In the time domain, the standard deviation of normal-to-normal intervals (SDNN) and the root mean square of standard deviation (RMSSD) will be examined. Additionally, low-frequency power (LF nu; bandwidth 0.04–0.15 Hz) and high-frequency power (HF nu; bandwidth 0.15–0.4 Hz), expressed as normalized units, as well as the LF nu/HF nu ratio will be examined as frequency-based HRV parameters. To the best of our knowledge, no MCID scores are currently available for HRV [126].

#### Perceived fitness

A one-item proxy measure is used to assess subjectively perceived physical fitness [127]. The following item will be used: “Overall, how would you rate your physical fitness?” Answering options range from 1 (very poor fitness) to 10 (excellent fitness). The validity of this item as an indicator of perceived fitness has been established previously. High correlations were found with the 12-item Perceived Physical Fitness scale [128]. Moreover, this measure proved to be reasonably associated with objective physical fitness, perceived well-being, and sleep [128, 129]. No MCID scores exist for this measure.

#### Exercise-related self-efficacy

Three items referring to beginning, maintaining, and restarting exercise after a relapse are used to assess exercise-related self-efficacy beliefs (e.g. “I feel confident to start with a new exercise activity”) [130]. This scale proved to be a psychometrically sound measure in a previous study [130] and has been used in previous intervention studies [80–82, 131]. The scale ranges from 0 (not at all confident) to 5 (100% confident in myself). The three items are added to obtain a single score. No MCID scores exist for this measure.

#### Exercise-related outcome expectancies

Nine positive (‘pros’; e.g. “I can improve my physical appearance if I regularly exercise”) and seven negative formulated items (‘cons’; e.g. “If I exercise, I end up in situations where I feel embarrassed”) are used to assess outcome expectancies [132]. The items are anchored on a 4-point Likert-type scale from 1 (not true) to 4 (completely true). Satisfactory psychometric properties of these items have been demonstrated [132]. Items are combined into two composite scores (positive and negative) by the arithmetic mean of each. No MCID scores exist for this measure.

#### Exercise intention

One item is used to assess exercise-related goal intentions [130]. More specifically, participants are asked about the strength of their intention to exercise regularly during the next few weeks and months (0 = no intention to 5 = very strong intention). This measure proved to have acceptable reliability and validity in previous studies [133, 134]. No MCID scores exist for this measure.

#### Exercise-related self-concordance

Exercise-related self-concordance is assessed with the 12-item SSK scale [133], which is consistent with the self-concordance model by Sheldon and Elliot [135]. The SSK scale consists of four subscales that assess the intrinsic (e.g. “I (would) exercise because it’s just fun for me”), identified (e.g. “I (would) exercise because I have good reasons to be physically active”), introjected (e.g. “I (would) exercise because otherwise I would have a guilty conscience”) and extrinsic (e.g. “I (would) exercise because others tell me to become physically active”) reasons for exercising. All items are answered on a 6-point Likert scale from 1 (not at all true) to 6 (completely true). An overall index is built by summing the identified and intrinsic mean scores and subtracting the introjected and extrinsic mean scores. The reliability and validity of this instrument have been established previously [133]. No MCID scores exist for this measure.

#### Action planning

Five items with established reliability and validity are administered to collect information about participants’ level of action planning [134, 136]. These items assess the degree to which individuals have pre-planned their exercise participation. Thus, participants are asked whether they normally make plans when, where, how, how often, and with whom they exercise. Answers range from 1 (not at all true) to 4 (completely true). The item scores are summed to obtain an overall index. No MCID scores exist for this measure.

#### Perceived exercise barriers

A 19-item scale that lists various obstacles to regular exercise participation is used to measure perceived exercise barriers [137]. Satisfactory psychometric properties of this instrument were reported previously [68, 137, 138]. Participants indicate on a 4-point Likert scale from 1 (almost never) to 4 (almost always) how often they perceive these barriers (e.g. “I have too much work to do”). The mean is computed to obtain a single score. No MCID scores exist for this measure.

#### Coping planning

A 5-item index is used to gain insight into coping planning [136]. Participants are asked to what degree they use self-regulation strategies to overcome potential exercise barriers (e.g. “I have made a detailed plan regarding what to do in difficult situations in order to act in according to my intentions”). Answers range from 1 (not at all true) to 4 (completely true). The item scores are summed up to obtain a composite index. The reliability and validity of this scale have been established previously [134, 136]. No MCID scores exist for this measure.

#### Exercise-related social support

A seven-item index is used to assess social support from relevant others (e.g. “Close family or friends help me plan my exercise”) [132]. Answers are given on a 4-point Likert scale with values from 1 (almost never) to 4 (almost always). This scale proved to be a psychometrically sound measure in previous studies [131, 132]. No MCID scores exist for this measure.

#### Depression severity

The HAMD17 total score [95] and the Beck Depression Inventory (BDI) [97, 98] are used to assess depression severity. The BDI is a 21-item tool frequently used to assess symptoms of unipolar depression such as affective, behavioural, cognitive, and somatic symptoms (e.g. “I am so unhappy/sad that I can’t stand it”). Four response options exist, which reflect increasing levels of depressive symptomatology. The HAMD17 and BDI total scores range from 0 to 52 and from 0 to 63, respectively, with higher scores reflecting stronger depressive symptoms. The reliability and validity of the HAMD17 [139, 140] and the BDI [141] are well documented in the scientific literature. Response and remission are defined based on the HAMD17 scores. Response is defined as ≥ 50% decrease of symptoms from baseline to the endpoint, partial response as 25–49% reduction, and non-response as < 25% reduction, whereas remission is accomplished if the HAMD17 total score is ≤ 7 [142]. With regard to the BDI, scores between 0 and 9 indicate that a person is not depressed, scores between 10 and 18 reflect mild–moderate depression, scores between 19 and 29 indicate moderate-to-severe depression, and scores ≥ 30 indicate severe depression [97]. Button et al. [143] further suggested for the BDI that the MCID is best measured on a ratio scale, with a reduction of 17.5% of the initial scale representing a clinically meaningful change in depressive symptoms.

#### Self-perceived stress

Perceived stress will be assessed with the Perceived Stress Scale (PSS) [144, 145]. The 10-item PSS is a well-established self-report measure of stress and is based on the cognitive-transactional stress theory. Answers are given on a 5-point Likert scale ranging from 1 (never) to 5 (very often). Evidence for the validity and reliability of the PSS has been reported previously [146]. No MCID scores exist for this measure.

#### Self-perceived health

The Medical Outcomes Study 12-Item Short Form Health Survey (SF-12) is used to assess participants’ self-perceived health [147]. This instrument is among the most widely used measures within general population research [148], and the reliability and validity of the SF-12 are well documented [149, 150]. The composite scores of both subscales (physical and psychological health) are calculated by weighting each item as described in the SF-12 manual. Higher scores reflect increased health functioning. A study with low back pain patients suggests that improvements of > 3.77 in the psychological subscale and of > 3.29 in the physical subscale can be regarded as suitable MCID scores [151].

#### Insomnia symptoms

Sleep complaints are assessed with the seven-item Insomnia Severity Index (ISI) [152]. Answers are given on a 5-point rating scale, ranging from 0 (not at all) to 4 (very much). Evidence for the reliability and validity of this instrument has been documented previously [153, 154]. These items are in line with DSM-IV criteria for insomnia and include symptoms such as difficulties falling asleep, difficulties maintaining sleep, early morning awakening, increased daytime sleepiness, low daytime performance, low satisfaction with sleep, and worrying about sleep. Higher scores reflect a higher level of sleep complaints. Scores of 0–7 indicate absence of insomnia, scores of 8–14 indicate subthreshold insomnia, scores of 15–21 indicate moderate insomnia, and scores of 22–28 indicate severe insomnia [153].

#### Cognitive function

Today, there is no gold standard regarding the assessment of cognitive and executive function [155]. With regard to cognitive function, we decided to apply an odd-ball paradigm, the two-back test, and the Flanker task to assess sustained attention, working memory, and inhibition, respectively. The odd-ball paradigm [156] requires participants to press a button to deviants, which appear with a lower frequency (25%) than standard stimuli (75%). In the two-back task [157], participants are instructed to identify whether or not the presented letter matches the one presented two trials before by pressing a button corresponding to yes or no. Lastly, the Flanker task [158] requires participants to respond to the direction of a centrally presented arrow and to ignore the flanking arrows, which either point in the same or the opposite direction. These computer-based cognitive tests are well recognized neuropsychological tests for assessing attention [159] and executive function [160], and have been found to be reliable tools in previous research [161–163]. In the present study, the cognitive tasks will be administered with E-Prime 3.0 (PST, USA). Separately for each test, the reaction time (on response-correct trials) and accuracy will be extracted for statistical analyses. No established MCID scores exist for this measure.

#### Blood pressure

Systolic (SBP) and diastolic (DBP) blood pressure will be measured after the participant has rested for 5 min while seated. Blood pressure is taken twice within 5 min with the Omron® digital blood pressure monitor. A cuff size appropriate to the arm circumference of the participants will be chosen. Evidence for the validity of Omron® oscillometric blood pressure measurement devices has been reported previously [164]. Reductions in SBP and DBP of 2 mmHg are considered meaningful MCID scores [165]. Participants will be considered hypertensive if they have SBP scores of ≥ 140 mmHg and DBP scores of ≥ 90 mmHg [166].

#### Body mass index, percentage of body fat, and waist circumference

Body weight will be measured with a digital weighing scale (BC-545; Tanita, USA) without shoes (to the nearest 0.1 kg, in light clothes). To measure height, each participant will stand against a stadiometer without shoes. Body height will be taken (to the nearest 0.5 cm). BMI will be calculated with the following formula: weight (kg) / (standing height (m))^2^. Based on WHO standards [167], participants will be classified as overweight if their BMI is ≥ 25.0 kg/m^2^, and as obese with a BMI of ≥ 30.0 kg/m^2^. Following an expert consensus [168], 5% weight loss is considered the MCID among people classified as overweight or obese. The BC-545 weighing scale can also be used for bioelectrical impedance analysis to assess percentage body fat. With regard to healthy body fat, the WHO [167] recommends maximum levels of ≥ 32% for women and ≥ 25% for men. In the present study, a reduction of 2% is defined as the MCID for percentage body fat [169]. A flexible tape at the natural waist (halfway between the ribcage and the iliac crest) is used to determine waist circumference. The expert panel of the National Cholesterol Education Program [170] defines a waist circumference of ≥ 80 cm (women) and ≥ 94 cm (men) as a risk factor for metabolic syndrome.

#### Cholesterol, triglycerides, and HbA1c

To perform blood tests, venous blood is drawn between 07:00 and 08:30 after fasting since 22:00 the day before by a trained nurse. For the assessment of blood lipid profiles (total cholesterol (TC), low-density-lipoprotein cholesterol (LDL-C), high-density-lipoprotein cholesterol (HDL-C), and triglycerides (TG)) and HbA1c, blood samples will be analysed via the Afinion test (Alere Technologies; Abbott, Wädenswil, Switzerland). One drop of blood will be taken up by the test strip and read by the machine. Good correspondence exists between the Alere point-of-care (PAC) analyser results and reference laboratory tests for HbA1c and lipid levels [171, 172]. Following Rodondi et al. [173], the following clinically relevant cut-off values should be considered for total cholesterol (≥ 5.6 mmol/l), HDL-C (≤ 1.41 mmol/l), and LDL-C (≥ 3.40 mmol/l). To our knowledge, no officially established MCID score exists for cholesterol levels, although some authors suggested considering a 10% increase in HDL-C and a 10% decrease in LDL-C as a meaningful score [174]. Following the American Diabetes Association [175] and the WHO [176], HbA1c scores of 5.7–6.4% point towards pre-diabetes, whereas scores of ≥ 6.5% can be used as diagnostic cut-off point for diabetes, and reductions of ≥ 0.5% are generally considered the MCID for type 2 diabetes [177].

#### Brain-derived neurotrophic factor

Serum BDNF levels [178] will be determined in duplicate with an enzyme-linked immunosorbent assay (BDNF Emax Immunoassay System; Promega, USA). This method showed intra- and inter-assay variation coefficients of 6.0 and 8.5%, respectively [179]. No MCID scores exist for this measure.

#### Tumour necrosis factor alpha

The solid-phase Enzyme Amplified Sensitivity Immunoassay TNF-α ELISA from DRG (Switzerland) will be used to assess TNF-α [178]. The assay uses monoclonal antibodies (mAbs) directed against distinct epitopes of TNF-α. The amount of substrate turnover is determined colourimetrically by measuring the absorbance, which is proportional to the TNF-α concentration. No MCID scores exist for this measure.

#### Insulin-like growth factor 1

The DRG IGF-I 600 ELISA Kit (DRG), a solid phase enzyme-linked immunosorbent assay (ELISA), based on the principle of competitive binding, will be used to assess IGF-1 [180]. Patient samples, standards, and controls are acidified and neutralized prior to the assay procedure. No MCID scores exist for this measure.

### Moderator

#### Serotonin transporter gene (5-HTTLPR)

To assess the 5-HTTLPR genotype [181] at baseline, 9 ml EDTA blood is collected from each participant (patients and controls). Within 2 h the blood sample is centrifuged without a break at 2500 × *g* for 10 min at room temperature to separate the cellular components. White blood cells are enriched in the resulting intermediate layer, which is carefully transferred to a reaction tube and stored at − 20 °C for no longer than 4 weeks prior to further processing. The next steps involve DNA extraction according to the manufacturer’s instructions using the QUIAamp DNA blood mini kit (Quiagen, Switzerland), 5-HTTLPR-specific DNA amplification using GoTaq Polymerase and the Promega PCR Master Mix (Promega), and the following 5-HTTLPR primers: forward, GGC GTT GCC GCT CTG AAT GC (annealing temperature 66 °C); and reverse, GAG GGA CTG AGC TGG ACA ACC AC (annealing temperature 74 °C). After completion of the PCR, the products are electrophoretically evaluated.

The four afore-mentioned biochemical and genetic analyses for BDNF, TNF-α, IGF-1, and 5-HTTLPR will be performed at the Neurobiological Laboratory of the Psychiatric University Clinics Basel (Prof. Anne Eckert).

### Covariates

The following variables will be assessed as potential covariates. Based on participants’ self-reports, we will assess their gender, age, language, nationality, marital status, level of education, employment (rate) prior to hospitalization, years of job experience, smoking status, and the number of children living at home. In addition, based on a clinical interview with the treating psychiatrist, we will assess information regarding duration of the current depressive episode, number of prior depressive episodes, age of onset of depression, and current medication.

### Qualitative data assessment

A nested qualitative study will be carried out using semi-structured interviews to assess the experience and acceptability of the intervention for the patients. All interviews will be tape-recorded, transcribed, and analysed with qualitative content analysis [182]. Moreover, the physical activity facilitator will register the number of hours needed for the counselling of each patient. As a result, we will be able to calculate the costs that need to be covered by a clinic if a trained professional is employed to facilitate physical activity adoption and maintenance. To assess cost-effectiveness of a physical activity intervention, Baxter et al. [87] and Chalder et al. [88] used a similar procedure in a previous study.

### Data collection and statistical analysis

Types of data to be collected include: quantitative data on social and demographic background, anthropometric measurements, physical activity, cardiorespiratory fitness, exercise-related variables (cognitive and social determinants), indicators of psychological functioning, cardiovascular risk factors, and biomarkers of MDD; and qualitative data, based on semi-structured interviews, on the acceptability and perceived usefulness of the intervention among the patients. The collected data will be entered and merged into a single datafile. Statistical analysis will be performed using SPSS.

Treatment group, age, gender, baseline HAMD17 score, and the amount of previous depressive episodes will be selected as covariables. Changes in outcome variables over the three time points will be analysed using repeated-measures analyses of covariance (rANCOVAs), with a between-subject factor group (IG vs PCG) and a within-subject factor time (baseline, post intervention, follow-up). If significant group or time interactions are present, Bonferroni-adjusted post-hoc tests will be performed to identify individual differences. The statistical significance level will be defined at α = 0.05. Effect sizes will be calculated according to the recommendations of Cohen [183], with 0.49 ≥ *d* ≥ 0.20 indicating small effect (e.g. negligible practical importance), 0.79 ≥ *d* ≥ 0.50 indicating medium effect (moderate practical importance), and *d* ≥ 0.80 indicating large effect (crucial practical importance).

In the case of missing values (e.g. when patients drop out before follow-up), all analyses will be performed with and without intention to treat [184]. After a thorough dropout analysis, a decision will be reached regarding the method best suited to analyse intention-to-treat effects (e.g. last observation carried forward, imputation of missing values) [185].

### Data monitoring and publishing of data

The sponsor-investigator will have monitoring visits at each site prior to the start and during the course of the intervention, to assure standardization across all four centres. Any observed discrepancies will be documented and further procedure discussed.

Furthermore, we will provide every fourth month a report to an external person (Prof. Uwe Pühse, Head Division Sport and Health Pedagogy, DSBG, University of Basel) regarding the recruitment quote, number of performed counselling sessions, state of the post-intervention and follow-up data assessments, and the dropout quote. In case of insufficient progress of the study, an agreement regarding potential counter-measures will be sought within the data monitoring committee (consisting of the external monitor (UP), the sponsor-investigator, and the responsible researchers of the four clinics (JB, MH, CI, UL)). The data monitoring committee will also coordinate the interim analysis and the international dissemination of the study results through presentations at national and international conferences and publications in peer-reviewed literature (primarily open access). The data monitoring committee will further decide which researchers (beyond those listed as co-authors in the present publication) will have access to the final trial dataset. In agreement with the other members of the data monitoring committee, the sponsor-investigator has the right to terminate the study prematurely according to certain circumstances, including ethical concerns, insufficient participant recruitment, when the safety of the participants is doubtful or at risk, respectively, alterations in accepted clinical practice that make the continuation of the trial unwise, and early evidence of benefit or harm of the experimental intervention.

At the end of the study, the results will be communicated to relevant healthcare professionals, the public, and other relevant institutions/groups.

### Safety

All serious adverse reactions (SAEs) and adverse events (AEs) that occur during the study will be immediately reported to the sponsor-investigator. More specifically, during the entire duration of the study, all AEs and all SAEs will be collected, fully investigated, and documented in source documents and case report forms. The study duration encompasses the time from when the participant signs the informed consent until the last protocol-specific procedure has been completed, including a safety follow-up period. The sponsor-investigator will ensure obtaining required insurance coverage for the trial under applicable laws.

### Schedule and milestones

Recruitment of patients started in January 2019. Each study centre treats approximately 150 patients per year. Assuming a recruitment rate of approximately 25%, inclusion will stop in January 2021, with follow-up assessments ending in January 2022. Recruitment of healthy controls started in January 2019 and will last until January 2020. An approximate schedule is presented in Table 1.

**Table 1:** Planned schedule and milestones

	2019				2020				2021				2022			
	1	2	3	4	1	2	3	4	1	2	3	4	1	2	3	4
Recruitment of patients	X	X	X	X	X	X	X	X	X							
Screening	X	X	X	X	X	X	X	X	X							
Baseline tests	X	X	X	X	X	X	X	X	X							
Implementation of intervention programme	X	X	X	X	X	X	X	X	X							
Post assessments		X	X	X	X	X	X	X	X	X						
Follow-up assessments					X	X	X	X	X	X	X	X	X			
Assessment of healthy controls	X	X	X	X	X											
Qualitative interviews		X	X	X	X	X	X	X	X							
Data analyses, writing-up results									X	X	X	X	X	X		

## Discussion

People with mental illnesses are especially vulnerable to cardiovascular and metabolic diseases primarily caused by a sedentary lifestyle. Individually tailored physical activity counselling may increase lifestyle physical activity and cardiorespiratory fitness in a particularly vulnerable population which often suffers from a mix of mental and physical health problems. Improving physical activity and cardiorespiratory fitness may have important implications for tackling metabolic and cardiovascular disease and increasing cognitive functioning in this at-risk population, hence reducing the future burden of multiple chronic conditions. Increased physical activity and cardiorespiratory fitness may also reduce the likelihood of future depressive episodes. By moving towards the primary prevention of chronic physical conditions, much can be done to enhance the quality and quantity of life of people with MDD.

These findings may strengthen the evidence for “exercise as medicine” as a holistic care option in routine clinical practice for people with MDD, by helping patients to adopt and maintain physically active lifestyles after the end of their hospital stay. The study can show feasible ways to achieve long-term behaviour change by integrating physical activity and exercise counselling in given clinical structures. Moreover, the study will show whether such an approach is acceptable for in-patients treated for MDD, and how much financial resources are needed to systematically implement lifestyle physical activity counselling.

### Trial status

The study protocol corresponds to the second protocol version, as submitted to the EKNZ and EKB on 28 September 2018. Recruitment started on 1 January 2019. Follow-up data assessment will be complete at the latest in January 2022. Ethical approval has been obtained from the relevant review boards in Switzerland.

## Additional file

Additional file 1: SPIRIT 2013 Checklist (DOC 123 kb)

## Data Availability

Data will be made publicly available as supplementary online material and stored in digital archives that correspond with FAIR Data Principals after publication of the data (in form of an SPSS file). Variables are clearly labelled in the SPSS file and described in the SPSS variable view. For each publication, a separate SPSS file will be created with the data used for the specific data analyses.

## Abbreviations

5-HTTLPR: Serotonin transporter polymorphic promoter region
ACSM: American College of Sports Medicine
AE: Adverse event
BASPO: Swiss Federal Office of Sport
BDI: Beck Depression Inventory
BDNF: Brain-derived neurotrophic factor
BMI: Body mass index
CRF: Cardiorespiratory fitness
DALY: Disease-adjusted life years
DBP: Diastolic blood pressure
DSM-IV: *Diagnostic and Statistical Manual of Mental Disorders* (4th edition)
ECG: Electrocardiogram
EKB: Ethikkommission Bern (Ethical Review Board of the Canton Bern)
EKNZ: Ethikkommission Nordwest- und Zentralschweiz (Ethical Review Board of Northwestern and Central Switzerland)
ELISA: Enzyme-linked immunosorbent assay
HAMD17: 17-item Hamilton Depression Rating Scale
HDL-C: High-density-lipoprotein cholesterol
HF nu: High-frequency normalized units
HRV: Heart rate variability
ICD-10: International Classification of Diseases (10th edition)
IG: Intervention group
IGF-1: Insulin-like growth factor 1
IPAQ: International Physical Activity Questionnaire
ISI: Insomnia Severity Index
LDL-C: Low-density-lipoprotein cholesterol
LF nu: Low-frequency normalized units
mAb: Monoclonal antibody
MDD: Major depressive disorder
MET: Metabolic equivalents of task
MoVo-Lisa: Motivation, Volition and Lifestyle-integrated Sport Activity
MPA: Moderate physical activity
PAR-Q: Physical Activity Readiness Questionnaire
PCG: Placebo control group
PSS: Perceived Stress Scale
rANCOVA: Repeated-measures analysis of covariance
RCT: Randomized controlled trial
R–R interval: Inter-beat interval for peak of the QRS complex of the ECG wave
SAE: Serious adverse event
SBP: Systolic blood pressure
SDNN: Standard deviation of normal-to-normal intervals
SF-12: 12-item Short Form Health Survey
SIMPAQ: Simple Physical Activity Questionnaire
SNSF: Swiss National Science Foundation
SPSS: Statistical Package for the Social Sciences
SSK: Exercise-related self-concordance
TC: Total cholesterol
TG: Triglycerides
TNF-α: Tumour necrosis factor alpha
VO_2_max: Maximal oxygen uptake
VPA: Vigorous physical activity
WHO: World Health Organization
YLD: Years of life lived with disability

## References

[1] WHO (2004). Prevention of mental disorders. Effective interventions and policy option: summary report.

[2] Kessler RC, Berglund P, Demler O, Jein R, Koretz D, Merikangas KR (2003). The epidemiology of major depressive disorder. Results from the National Comorbidity Survey Replication. JAMA..

[3] Kessler RC, Berglund P, Demler O, Jin R, Merikangas KR, Walters EE (2005). Lifetime prevalence and age-of-onset distributions of DSM-IV disorders in the national comorbidity survey replication. Arch Gen Psychiatry.

[4] Blumenthal JA, Michael A, Babjak A (2007). Exercise and pharmacotherapy in the treatment of major depressive disorder. Psychosom Med.

[5] Murray CJL, Lopez AD (1997). Global mortality, disability, and the contribution of risk factors: global burden of disease study. Lancet..

[6] Lépine J-P, Briley M (2011). The increasing burden of depression. Neuropsychiatr Dis Treat.

[7] WHO (2001). The World Health Report 2001—mental health: new understanding, new hope.

[8] Blumenthal JA, Smith PJ, Hofmann BM (2012). Is exercise a viable treatment for depression?. ACSMs Health Fit J.

[9] Vancampfort D, Mitchell AJ, Hert M, Sienaert P, Probst M, Buys R (2015). Type 2 diabetes in patients with major depressive disorder: a meta-analysis of prevalence estimates and predictors. Depress Anxiety.

[10] Luppino FS, de Wit LM, Bouvy PF, Stijnen T, Cuijpers P, Penninx BW (2010). Overweight, obesity, and depression: a systematic review and meta-analysis of longitudinal studies. Arch Dis Child.

[11] Pan A, Sun Q, Okereke OI, Rexrode KM, Hu FB (2011). Depression and risk of stroke morbidity and mortality: a meta-analysis and systematic review. J Am Med Assoc.

[12] Dhar AK, Baron DA. Depression and the link with cardiovascular disease. Front Psychiatry. 2016;7. 10.3389/fpsyt.2016.00033. 10.3389/fpsyt.2016.00033 PMC4800172 27047396

[13] Vancampfort D, Correll CU, Wampers M, Sienaert P, Mitchell AJ, De Herdt A (2014). Metabolic syndrome and metabolic abnormalities in patients with major depressive disorder: a meta-analysis of prevalences and moderating variables. Psychol Med.

[14] Dishman RK, Berthoud H, Booth F, Cotman CW, Edgerton VR, Fleshner M (2006). Neurobiology of exercise. Obesity..

[15] Meltzer H (1989). Serotonergic dysfunction in depression. Br J Psychiatry.

[16] Goodnick PJ, Goldstein BI (1998). Selective serotonin reuptake inhibitors in affective disorders—II. Efficacy and quality of life. J Psychopharmacol.

[17] Caspi A, Sugden K, Moffitt TE, Taylor A, Craig IW, Harrington HL (2003). Influence of life stress on depression: moderation by a polymorphism in the 5-HTT gene. Science..

[18] Lesch KP, Bengel D, Heils A, Sabol SZ, Greenberg BD, Petri S (1996). Association of anxiety-related traits with a polymorphism in the serotonin transporter gene regulatory region. Science..

[19] Risch N, Herrell R, Lehner T, Liang KY, Eaves L, Hoh J (2009). Interaction between the serotonin transporter gene (5-HTTLPR), stressful life events, and risk of depression: a meta-analysis. JAMA..

[20] Munafò MR, Durrant C, Lewis G, Flint J (2009). Gene x environment interactions at the serotonin transporter locus. Biol Psychiatry.

[21] Rethorst CD, Landers DM, Nagoshi CT, Ross JT (2011). The association of 5-HTTLPR genotype and depressive symptoms is moderated by physical activity. J Psychiatr Res.

[22] Cuijpers P, Vogelzangs N, Twisk JW, Kleiboer A, Li J, Penninx BW (2014). Differential mortality rates in major and subthreshold depression: meta-analysis of studies that measured both. Br J Psychiatry.

[23] Walker ER, McGee RE, Druss BG (2015). Mortality in mental disorders and global disease burden implications: a systematic review and meta-analysis. JAMA Psychiatry.

[24] Lawrence D., Hancock K. J., Kisely S. (2013). The gap in life expectancy from preventable physical illness in psychiatric patients in Western Australia: retrospective analysis of population based registers. BMJ.

[25] Thornicroft G (2011). Physical health disparities and mental illness: the scandal of premature mortality. Br J Psychiatry.

[26] Farah WH, Alsawas M, Mainou M, Alahdab F, Farah MH, Ahmed AT (2016). Non-pharmacological treatment of depression: a systematic review and evidence map. Evid Based Med.

[27] Salehi I, Hosseini SM, Haghighi M, Jahangard L, Bajoghli H, Gerber M (2016). Electroconvulsive therapy (ECT) and aerobic exercise training (AET) increased plasma BDNF and ameliorated depressive symptoms in patients suffering from major depressive disorder. J Psychiatr Res.

[28] Mota-Pereira J, Silverio J, Carvalho S, Ribeiro JC, Fonte D, Ramos J (2011). Moderate exercise improves depression parameters in treatment-resistant patients with major depressive disorder. J Psychiatr Res.

[29] Jahangard L, Sadeghi A, Ahmadpanah M, Holsboer-Trachsler E, Sadeghi Bahmani D, Haghighi M (2018). Influence of adjuvant omega-3-polyunsaturated fatty acids on depression, sleep, and emotion regulation among outpatients with major depressive disorders—results from a double-blind, randomized and placebo-controlled clinical trial. J Psychiatr Res.

[30] Olfson M, Marcus SC, Druss B, Elinson L, Tanielian T, Pincus HA (2002). National trends in the outpatient treatment of depression. JAMA..

[31] Blumenthal JA, Babyak MA, Moore KA, Craighead WE, Herman S, Khatri P (1999). Effects of exercise training on older patients with major depression. Arch Intern Med.

[32] Bauer M, Pfennig A, Severus E, Whybrow PC, Angst J, Möller H-J (2013). World Federation of Societies of Biological Psychiatry. Task Force on Unipolar Depressive Disorders. World Federation of Societies of Biological Psychiatry (WFSBP) guidelines for biological treatment of unipolar depressive disorders, part 1: update 2013 on the acute and continuation treatment of unipolar depressive disorders. World J Biol Psychiatry.

[33] Knapen Jan, Vancampfort Davy, Moriën Yves, Marchal Yannick (2014). Exercise therapy improves both mental and physical health in patients with major depression. Disability and Rehabilitation.

[34] Waring WS (2012). Clinical use of antidepressant therapy and associated cardiovascular risk. Drug Healthcare Patient Safety.

[35] Lingam R, Scott J (2002). Treatment non-adherence in affective disorders. Acta Psychiatr Scand.

[36] Smith D, Dempster C, Glanville J, Feemantle N, Anderson I (2002). Efficacy and tolerability of venlafaxine compared with selective serotonin re-uptake inhabitors and other antidepresssants: a meta-analysis. Br J Psychiatry.

[37] Trivedi MH, Daly EJ (2008). Treatment strategies to improve and sustain remission in major depressive disorder. Dialogues Clin Neurosci.

[38] Nemeroff CB (2007). Prevalence and management of treatment-resistant depression. J Clin Psychiatry.

[39] Trivedi MH, Fava M, Wisniewski M, Thase ME, Quitkin F, Warden D (2006). Medication augmentation after the failure of SSRIs for depression. N Engl J Med.

[40] Rethorst CD, Wipfli BM, Landers DM (2009). The antidepressive effects of exercise: a meta-analysis of randomized trials. Sports Med.

[41] Kirsch I, Deacon BJ, Huedo-Medina TB, Scoboria A, Moore TJ, Johnson BT (2008). Initial severity and antidepressant benefits: a meta-analysis of data submitted to the food and drug administration. PLoS Med.

[42] Cassano P, Fava M (2002). Depression and public health—an overview. Psychosom Res.

[43] Farnia V, Hojatitabar S, Shakeri J, Rezaei M, Yazdchi K, Bajoghli H (2015). Adjuvant Rosa damascena has a small effect on SSRI-induced sexual dysfunction in female patients suffering from MDD. Pharmacopsychiatry..

[44] Farnia V, Shirzadifar M, Shakeri J, Rezaei M, Bajoghli H, Holsboer-Trachsler E (2015). Rosa damascena oil improves SSRI-induced sexual dysfunction in male patients suffering from major depressive disorders: results from a double-blind, randomized, and placebo-controlled clinical trial. Neuropsychiatr Dis Treat.

[45] Alvarez-Jiménez M, Conzalez-Blanch C, Vazquez-Barquero JL, Pérez-Iglesias R, Martinez-Garcia O, Pérez-Pardal T (2006). Attenuation of antipsychotic-induced weight gain with early behavioral intervention in drug-naive first-episode psychosis patients: a randomized controlled trial. J Clin Psychiatry.

[46] Kessler RC, Soukup J, Davis RB, Foster DF, Wilkey SA, Van Rompay MI (2001). The use of complementary and alternative therapies to treat anxiety and depression in the United States. Am J Psychiatr.

[47] Astin JA (1998). Why patients use alternative medicine—results of a national study. JAMA..

[48] Dunn AL, Trivedi MH, Kampert JB, Clark CG, Chambliss HO (2005). Exercise treatment for depression. Efficacy and dose response. Am J Prev Med.

[49] Lawlor DA, Hopker SW (2005). The effectiveness of exercise as intervention in the management of depression: systematic review and meta-regression analysis of randomised controlled trials. Br Med J.

[50] Stubbs Brendon, Vancampfort Davy, Hallgren Mats, Firth Joseph, Veronese Nicola, Solmi Marco, Brand Serge, Cordes Joachim, Malchow Berend, Gerber Markus, Schmitt Andrea, Correll Christoph U., De Hert Marc, Gaughran Fiona, Schneider Frank, Kinnafick Florence, Falkai Peter, Möller Hans-Jürgen, Kahl Kai G. (2018). EPA guidance on physical activity as a treatment for severe mental illness: a meta-review of the evidence and Position Statement from the European Psychiatric Association (EPA), supported by the International Organization of Physical Therapists in Mental Health (IOPTMH). European Psychiatry.

[51] Mammen G, Faulkner G (2013). Physical activity and the prevention of depression: a systematic review of prospective studies. Am J Prev Med.

[52] Gerber Markus, Minghetti Alice, Beck Johannes, Zahner Lukas, Donath Lars (2019). Is improved fitness following a 12-week exercise program associated with decreased symptom severity, better wellbeing, and fewer sleep complaints in patients with major depressive disorders? A secondary analysis of a randomized controlled trial. Journal of Psychiatric Research.

[53] Cooney GM, Dwan K, Greig CA, Lawlor DA, Rimer J, Waugh FR, et al. Exercise for depression. Cochrane Database Syst Rev. 2013;(9):CD004366. 10.1002/14651858.CD004366.pub6. 10.1002/14651858.CD004366.pub6 PMC9721454 24026850

[54] Schuch FB, Vancampfort D, Richards J, Rosenbaum S, Ward PB, Stubbs B (2016). Exercise as a treatment for depression: a meta-analysis adjusting for publication bias. J Psychiatr Res.

[55] Kvam S, Kleppe CL, Nordhus IH, Hovland A (2016). Exercise as a treatment for depression: a meta-analysis. J Affect Disord.

[56] Nebiker L, Lichtenstein E, Minghetti A, Zahner L, Gerber M, Faude O, et al. Moderating effects of exercise duration and intensity in neuromuscular versus endurance exercise interventions for the treatment of depression: a meta-analytical review with meta-regression and sensitivity analyses. Front Psychiatry. 2018;9(305). 10.3389/fpsyt.2018.00305. 10.3389/fpsyt.2018.00305 PMC6060256 30072923

[57] Minghetti A, Faude O, Hanssen H, Zahner L, Gerber M, Donath L (2018). Sprint interval training (SIT) substantially reduces depressive symptoms in major depressive disorder (MDD): a randomized controlled trial. Psychiatry Res.

[58] Gerber M, Minghetti A, Beck J, Zahner L, Donath L. Sprint interval training and continuous aerobic exercise training have similar effects on exercise motivation and affective responses to exercise in patients with major depressive disorders: a randomized controlled trial. Front Psychiatry. 2018. 10.3389/fpsyt.2018.00694. 10.3389/fpsyt.2018.00694 PMC6308196 30622487

[59] Hoffman BM, Babyak MA, Craighead E, Sherwood A, Doraiswamy PM, Coons MJ (2011). Exercise and pharmacotherapy in patients with major depression: one-year follow-up of the SMILE study. Psychosom Med.

[60] Babyak M, Blumenthal JA, Herman S, Khatri P, Doraiswamy M, Moore KA (2000). Exercise treatment for major depression: maintenance of therapeutic benefit at 10 months. Psychosom Med.

[61] HWr K, Murray T (2012). Foundations of physical activity and public health. Champaign: Human Kinetics.

[62] Blair SN, Kohl HW, Barlow CE, Paffenbarger RS, Gibbons LW, Macera CA (1995). Changes in physical fitness and all-cause mortality. A prospective study of healthy and unhealthy men. JAMA..

[63] Kodama S, Saito K, Tanaka S, Maki M, Yachi Y, Asumi M (2009). Cardiorespiratory fitness as a quantitative predictor of all-cause mortality and cardiovascular events in healthy men and women: a meta-analysis. JAMA..

[64] Lindwall M, Gerber M, Jonsdottir I, Börjesson M, Ahlborg GJ (2014). The relationships of change in physical activity with change in depression, anxiety, and burnout: a longitudinal study of Swedish healthcare workers. Health Psychol.

[65] Gerber M, Jonsdottir IH, Lindwall M, Ahlborg G (2014). Physical activity in employees with differing occupational stress and mental health profiles: a latent profile analysis. Psychol Sport Exerc.

[66] Gerber M, Lindwall M, Lindegård A, Börjesson M, Jonsdottir IH (2013). Cardiovascular fitness protects from stress-related symptoms of burnout and depression. Patient Educ Couns.

[67] Voderholzer U, Dersch R, Dickhut HH, Herter A, Freyer T, Berger M (2011). Physical fitness in depressive patients and impact of illness course and disability. J Affect Disord.

[68] Krämer LV, Helmes AW, Seelig H, Fuchs R, Bengel J (2014). Correlates of reduced exercise behaviour in depression: the role of motivational and volitional deficits. Psychol Health.

[69] Firth J, Rosenbaum S, Stubbs B, Gorczynski P, Yung AR, Vancampfort D (2016). Motivationg factors and barriers towards exercise in severe mental illness: a systematic review and meta-analysis. Psychol Med.

[70] Stubbs B, Rosenbaum S, Vancampfort D, Ward PB, Schuch FB (2016). Exercise improves cardiorespiratory fitness in people with depression: a meta-analysis of randomized controlled trials. J Affect Disord.

[71] Knapen J, Vancampfort D (2013). Evidence for exercise therapy in the treatment of depression and anxiety. Int J Psychosoc Rehabil.

[72] Schuch FB, Vasconcelos-Moreno MP, Borowsky C, Zimmerman AB, Rocha NS, Fleck MP (2015). Exercise and severe major depression: effect on symptom severity and quality of life at discharge in an inpatient cohort. J Psychiatr Res.

[73] Lindegård A, Jonsdottir IH, Börjesson M, Lindwall M, Gerber M. Changes in mental health in compliers and non-compliers with physical activity recommendations in patients with stress-related exhaustion. BMC Psychiatry. 2015;15. 10.1186/s12888-015-0642-3. 10.1186/s12888-015-0642-3 PMC4632342 26530329

[74] Pomp S, Fleig L, Schwarzer R, Lippke S (2012). Depressive symptoms interfere with post-rehabilitation exercise: outcome expectancies and experience as mediators. Psychol Health Med.

[75] Vancampfort D, Stubbs B, Sienaert P, Wyckaert S, De Hert M, Rosenbaum S (2015). What are the factors that influence physical activity participation in individuals with depression? A review of physical activity correlates from 59 studies. Psychiatr Danub.

[76] Gerber M, Holsboer-Trachsler E, Pühse U, Brand S (2016). Exercise is medicine for patients with major depressive disorders. But only if the “pill” is taken!. Neuropsychiatr Dis Treat.

[77] Gerber M, Holsboer-Trachsler E, Pühse U, Brand S (2016). Author’s reply to the Letter to the Editor “Exercise is medicine for depression: Even if the “pill” is small” of Hallgren, Stubbs and Vancampfort. Neuropsychiatr Dis Treat.

[78] Nigg CR (2013). ACSM's behavioral aspects of physical activity and exercise.

[79] Schwarzer R, Lippke S, Luszczynska A (2011). Mechanisms of health behavior change in persons with chronic illness or disability: the Health Action Process Approach (HAPA). Rehabil Psychol.

[80] Fuchs R, Göhner W, Seelig H (2011). Long-term effects of a psychological group intervention on physical exercise and health: the MoVo concept. J Phys Act Health.

[81] Fuchs R, Göhner W, Seelig H (2011). Effects of a standardized group intervention on physical exercise and health: the MoVo-concept. J Phys Act Health.

[82] Gerber M, Fuchs R, Pühse U (2010). Der Einfluss eines Kurz-Interventionsprogramms (MoVo-Lisa) auf das Bewegungsverhalten und die Determinanten köperlich-sportlicher Aktivität bei übergewichtigen und fettleibigen Personen [Effects of a short exercise-intervention on sport participation and cognitive-behavioral antecedents of sport participation in a sample of overweight and obese individuals]. Zeitschrift für Gesundheitspsychologie..

[83] Sniehotta FF, Scholz U, Schwarzer R, Fuhrmann B, Kiwus U, Voller H (2005). Long-term effects of two psychological interventions on physical exercise and self-regulation following coronary rehabilitation. Int J Behav Med.

[84] Lippke S, Ziegelmann JP, Schwarzer R (2004). Behavioral intentions and action plans promote physical exercise: a longitudinal study with orthopedic rehabilitation patients. J Sport Exerc Psychol.

[85] Garcia-Toro M, Ibarra O, Gili M, Serrano MJ, Olivan B, Vicens E (2012). Four hygienic-dietary recommendations as add-on treatment in depression: a randomized-controlled trial. J Affect Disord.

[86] Chalder M, Wiles NJ, Campbell J, Hollinghurst SP, Haase AM, Taylor AH (2012). Facilitated physical activity as a treatment for depressed adults: randomized controlled trial. BMJ..

[87] Baxter H, Winder R, Chalder M, Wright C, Sherlock S, Haase A (2010). Physical activity as a treatment for depression: the TREAD randomised trial protocol. Trials..

[88] Chalder M, Wiles NJ, Campbell J, Hollinghurst SP, Searle A, Haase A, et al. A pragmatic randomised controlled trial to evaluate the cost-effectiveness of a physical activity intervention as a treatment for depression: the TREAting Depression with physical activity (TREAD) trial. Health Technol Assess. 2012;16. 10.3310/hta16100. 10.3310/hta16100 22398106

[89] Rosenbaum S, Newby J, Steel Z, Andrews G, Ward PB (2015). Online physical activity interventions for mental disorders: a systematic review. Internet Interv.

[90] Haskell WL, Blair SN, Hill JO (2009). Physical activity: health outcomes and importance for public health policy. Prev Med.

[91] Haskell WL, Lee IM, Pate RR, Powell WW, Blair SN, Franklin BA (2007). Updated recommendation for adults from the American College of Sports Medicine and the American Heart Association. Med Sci Sport Exerc.

[92] Hillsdon M, Foster C, Thorogood M. Interventions for promoting physical activity. Cochrane Db Syst Rev. 2005;25:CD003180. 10.1002/14651858.CD003180.pub2 PMC4164373 15674903

[93] Foster C, Richards J, Thorogood M, Hillsdon M (2013). Remote and web 2.0 interventions for promoting physical activity. Cochrane Database Syst Rev.

[94] Stubbs B, Vancampfort D, Rosenbaum S, Ward PB, Richards J, Soundy A (2016). Dropout from exercise randomized controlled trials among people with depression: a meta-analysis and meta regression. J Affect Disord.

[95] Hamilton M (1967). Development of a rating scale for primary depressive illness. Br J Soc Clin Psychol.

[96] Williams JB (1988). A structured interview guide for the Hamilton Depression Rating scale. Arch Gen Psychiatry.

[97] Beck AT, Steer RA, Carbin MG (1988). Psychometric properties of the Beck Depression Inventory: twenty-five years of evaluation. Clin Psychol Rev.

[98] Beck AT, Ward CH, Mendelson M, Mock J, Erbaugh J (1961). An inventory for measuring depression. Arch Gen Psychiatry.

[99] Craig CL, Marshall AL, Sjöström M, Bauman AE, Booth ML, Ainsworth BE (2003). International Physical Activity Questionnaire: 12-country reliability and validity. Med Sci Sports Exerc.

[100] Thomas S, Reading J, Shephard RJ (1992). Revision of the Physical Activity Readiness Questionnaire (PAR-Q). Can J Sport Sci.

[101] SGAD, SGBP, SGPP (2016). Die somatische Behandlung der unipolaren depressiven Störungen: Update 2016. Swiss Medical Forum.

[102] Michie S, Richardson M, Johnston M, Abraham C, Francis J, Hardeman W (2013). The behavior change technique taxonomy (v1) of 93 hierarchically clustered techniques: building an international consensus for the reporting of behavior change interventions. Ann Behav Med.

[103] Michie S, van Stralen MM, West R. The behaviour change wheel: a new method for characterising and designing behaviour change interventions. Implement Sci. 2011;6. 10.1186/748-5908-6-42. 10.1186/1748-5908-6-42 PMC3096582 21513547

[104] Michie S, Abraham C, Whittington C, McAteer J, Gupta S (2009). Effective techniques in healthy eating and physical activity interventions: a meta-regression. Health Psychol.

[105] Samdal GB, Eide GE, Barth T, Williams G, Meland E. Effective behaviour change techniques for physical activity and healthy eating in overweight and obese adults: systematic review and meta-regression analyses. Int J Behav Nutr Phys Act. 2017;14(42). 10.1186/s12966-017-0494-y. 10.1186/s12966-017-0494-y PMC5370453 28351367

[106] Howlett Neil, Trivedi Daksha, Troop Nicholas A, Chater Angel Marie (2018). Are physical activity interventions for healthy inactive adults effective in promoting behavior change and maintenance, and which behavior change techniques are effective? A systematic review and meta-analysis. Translational Behavioral Medicine.

[107] Olander EK, Fletcher H, Williams S, Atkinson L, Turner A, French DP. What are the most effective techniques in changing obese individuals’ physical activity self-efficacy and behaviour: a systematic review and meta-analysis. Int J Behav Nutr Phys Act. 2013;10(29). 10.1186/479-5868-10-29. 10.1186/1479-5868-10-29 PMC3639155 23452345

[108] Krämer L, Helmes AW, Bengel J (2014). Understanding activity limitations in depression: integrating the concepts of motivation and volition from health psychology into clinical psychology. Eur Psychol.

[109] Marcus DK, O'Connell D, Norris AL, Sawaqdeh A (2014). Is the Dodo bird endangered in the 21st century? A meta-analysis of treatment comparison studies. Clin Psychol Rev.

[110] Wampold BE, Mondin GW, Moody M, Stich F, Benson K, Ahn H-N (1997). A meta-analysis of outcome studies comparing bona fide psychotherapies: empiricially, “all must have prizes”. Psychol Bull.

[111] Rowlands AV (2007). Accelerometer assessment of physical activity in children: an update. Pediatr Exerc Sci.

[112] Freedson PS, Melanson E, Sirard J (1998). Calibration of the Computer Science and Applications, Inc. accelerometer. Med Sci Sports Exerc.

[113] Ainsworth BE, Haskell WL, Whitt MC, Irwin ML, Swartz AM, Strath SJ, et al. Compendium of Physical Sctivities: an update of activity codes and MET intensities. Med Sci Sports Exerc. 2000;32(9):S498–S516. 10.1097/00005768-200009001-00009 10993420

[114] Ainsworth BE, Haskell WL, Herrmann SD, Meckes N, Bassett DR, Tudor-Locke C (2011). 2011 Compendium of Physical Activities: a second update of codes and MET values. Med Sci Sports Exerc.

[115] Trost SG, Pate RR, Freedson PS, Sallis JF, Taylor WC (2000). Using objective physical activity measures with youth: how many days of monitoring are needed?. Med Sci Sport Exerc..

[116] Herrmann SD, Barreira TV, Kang M, Ainsworth BE (2013). How many hours are enough? Accelerometer wear time may provide bias in daily activity estimates. J Phys Act Health.

[117] Herman Hansen B, Børtnes I, Hildebrand M, Holme I, Kolle I, Anderssen SA (2014). Validity of the Actigraph GT1M during walking and cycling. J Sports Sci.

[118] Rosenbaum Simon, Ward Philip B (2016). The Simple Physical Activity Questionnaire. The Lancet Psychiatry.

[119] Schilling René, Schärli Eveline, Fischer Xenia, Donath Lars, Faude Oliver, Brand Serge, Pühse Uwe, Zahner Lukas, Rosenbaum Simon, Ward Philip B., Carraro Attilio, Gerber Markus (2018). The utility of two interview-based physical activity questionnaires in healthy young adults: Comparison with accelerometer data. PLOS ONE.

[120] Åstrand P-O, Rodahl K (2003). Textbook of work physiology: physiological bases of exercise.

[121] Borg G (1970). Perceived exertion as an indicator of somatic stress. Scand J Rehabil Med.

[122] Macsween A (2001). The reliability and validity of the Astrand nomogram and linear extrapolation for deriving VO2max from submaximal exercise data. J Sports Med Phys Fitness.

[123] Ernst G. Hidden signals—the history and methods of heart rate variability. Front Public Health. 2017;5. 10.3389/fpubh.2017.00265. 10.3389/fpubh.2017.00265 PMC5649208 29085816

[124] Tarvainen MP, Niskanen JP, Lipponen JA, Ranta-Aho PO, Karjalainen PA (2014). Kubios HRV—heart rate variability analysis software. Comput Methods Prog Biomed.

[125] Giles D, Draper N, Neil W (2016). Validity of the Polar V800 heart rate monitor to measure RR intervals at rest. Eur J Appl Physiol.

[126] Bassi D, Santos-de-Araujo AD, Camargo PF, Dibai-Filho AV, da Fonseca MA, Mendes RG, et al. Inter and intra-rateer reliability of short-term measurement of heart rate variability on rest in diabetic type 2 patients. J Med Syst. 2018:42. 10.1007/s10916-018-1101-8, 10.1007/s10916-018-1101-8. 10.1007/s10916-018-1101-8 30327942

[127] Plante TG, LeCaptain SE, McLain HC (2000). Perceived fitness predicts daily coping better than physical activity. J Appl Biobehav Res.

[128] Plante TG, Lantis A, Checa G (1998). The influence of perceived versus aerobic fitness on psychological health and physiological stress responsitivity. Int J Stress Manag.

[129] Gerber M, Brand S, Holsboer-Trachsler E, Pühse U (2010). Fitness and exercise as correlates of sleep complaints. Is it all in our minds?. Med Sci Sports Exerc.

[130] Fuchs R (2008). Aufbau eines körperlich-aktiven Lebensstils im Kontext der medizinischen Rehabilitation: Ein motivational-volitionales Interventionskonzept (MoVo-LISA Projekt). Unveröffentlichter Endbericht.

[131] Gerber M, Fuchs R, Pühse U (2010). Follow-up of a short motivational and volitional exercise-intervention trial with overweight and obese individuals. Schweizerische Zeitschrift für “Sportmedizin und Sporttraumatologie”.

[132] Fuchs R (1997). Psychologie und körperliche Bewegung [Psychology and physical activity].

[133] Seelig H, Fuchs R (2006). Messung der sport- und bewegungsbezogenen Selbstkonkordanz [Measurement of sport and exercise related self-concordance]. Z Sportpsychol.

[134] Gerber M, Mallett C, Pühse U (2011). Beyond intentional processes: the role of action and coping planning in explaining exercise behaviour among adolescents. Int J Sport Exerc Psychol.

[135] Sheldon KM, Elliot AJ (1999). Goal striving, need-satisfaction, and longitudinal well-being: the self-concordance model. J Pers Soc Psychol.

[136] Sniehotta FF, Schwarzer R, Scholz U, Schüz B (2005). Action planning and coping planning for long-term lifestyle change: theory and assessment. Eur J Soc Psychol.

[137] Krämer L, Göhner W, Seelig H, Fuchs R, Sudeck G, Conzelmann A, Lehnert K, Gerlach E (2008). Barrieren und Barrierenmanagement im Prozess der Sportteilnahme. Differentielle Sportpsychologie—Sportwissenschaftlich Persönlichkeitsforschung 40 Jahrestagung der Arbeitsgemeinschaft für Sportpsychologie (asp) vom 1-3 Mai in Bern.

[138] Krämer L, Fuchs R (2010). Barrieren und Barrierenmanagement im Prozess der Sportteilnahme: Zwei neue Messinstrumente. Zeitschrift für Gesundheitspsychologie.

[139] Fava GA, Kellner R, Munari F, Pavan L (1982). The Hamilton Depression Rating Scale in normals and depressives. Acta Psychiatr Scand.

[140] Endicott J, Cohen J, Nee J, Fleiss J, Sarantakos S (1981). Hamilton Depression Rating Scale extracted from regular and change versions of the Schedule for Affective Disorders and Schizophrenia. Arch Gen Psychiatry.

[141] Richter P, Werner J, Heerlein A, Kraus A, Sauer H (1998). On the validity of the Beck Depression Inventory. Psychopathology..

[142] Wollmer MA, de Boer C, Kalak N, Beck J, Gotz T, Schmidt T (2012). Facing depression with botulinum toxin: a randomized controlled trial. J Psychiatr Res.

[143] Button KS, Kounali D, Thomas L, Wiles NJ, Peters TJ, Welton NJ (2015). Minimal clinically important difference on the Beck Depression Inventory—II according to the patient's perspective. Psychol Med.

[144] Cohen S, Kamarck T, Mermelstein R (1983). A global measure of perceived stress. J Health Soc Behav.

[145] Cohen S, Williamson GM, Spacapan S, Oskamp S (1988). Perceived stress in a probability sample of the united states. The social psychology of health.

[146] Klein EM, Brähler E, Dreier M, Reinecke L, Müller KW, Schmutzer G, et al. The German version of the Perceived Stress Scale—psychometric characteristics in a representative German community sample. BMC Psychiatry. 2016;16. 10.1186/s12888-016-0875-9. 10.1186/s12888-016-0875-9 PMC4877813 27216151

[147] Bullinger M, Kirchberger I (2011). SF-36 Fragebogen zum Gesundheitszustand.

[148] Rejeski WJ, Brawley LR, Shumaker SA (1996). Physical activity and health-related quality of life. Exerc Sport Sci Rev.

[149] Salyers MP, Bosworth HB, Swanson JW, Lamb-Pagone J, Osher FC (2000). Reliability and validity of the SF-12 Health Survey among people with severe mental illness. Med Care.

[150] Luo X, George ML, Kakouras I, Edwards CL, Pietrobon R, Richardson W (2003). Reliability, validity, and responsiveness of the short form 12-item survey (SF-12) in patients with back pain. Spine..

[151] Díaz-Arribas MJ, Fernández-Serrano M, Royuela A, Kovacs FM, Gallego-Izquierdo T, Ramos-Sánchez M (2017). Minimal clinically important difference in quality of life for patients with low back pain. Spine..

[152] Bastien CH, Vallières A, Morin CM (2001). Validation of the Insomnia Severity Index (ISI) as an outcome measure for insomnia research. Sleep Med.

[153] Morin CM, Belleville G, Belanger L, Ivers H (2011). The Insomnia Severity Index: psychometric indicators to detect insomnia cases and evaluate treatment response. Sleep..

[154] Gerber M, Lang C, Lemola S, Colledge F, Kalak N, Holsboer-Trachsler E, et al. Validation of the German version of the Insomnia Severity Index in adolescents, young adults and adult workers: results from three cross-sectional studies. BMC Psychiatry. 2016;16. 10.1186/s12888-016-0876-8. 10.1186/s12888-016-0876-8 PMC4888604 27245844

[155] Chan RC, Shum D, Toulopoulou T, Chen EY (2008). Assessment of executive functions: review of instruments and identification of critical issues. Arch Clin Neuropsychol.

[156] Huettel SA, McCarthy G (2004). What is odd in the oddball task? Prefrontal cortex is activated by dynamic changes in response strategy. Neuropsychologia..

[157] Mackworth JF (1959). Paced memorizing in a continuous task. J Exp Psychol.

[158] Eriksen BA, Eriksen CW (1974). Effects of noise letters upon the identification of a target letter in a nonsearch task. Percept Psychophys.

[159] García-Larrea L, Lukaszewicz AC, Mauguiére F (1992). Revisiting the oddball paradigm. Non-target vs neutral stimuli and the evaluation of ERP attentional effects. Neuropsychologia..

[160] Diamond A (2013). Executive functions. Annu Rev Psychol.

[161] Soveri A, Lehtonen M, Karlsson LC, Lukasik K, Antfolk J, Laine M (2018). Test–retest reliability of five frequently used executive tasks in healthy adults. Appl Neuropsychol.

[162] Wöstmann NM, Aichert DS, Costa A, Rubia K, Möller HJ, Ettinger U (2013). Reliability and plasticity of response inhibition and interference control. Brain Cogn.

[163] Williams LM, Simms E, Clark CR, Paul RH, Rowe D, Gordon E (2005). The test-retest reliability of a standardized neurocognitive and neurophysiological test battery:“neuromarker”. Int J Neurosci.

[164] Ostchega Y, Nwankwo T, Sorlie PD, Wolz M, Zipf G (2009). Assessing the validity of the Omron HEM-907XL oscillometric blood pressure measurement device in a national survey environment. J Clin Hypertens.

[165] Cook NR, Cohen J, Hebert PR, Taylor JO, Hennekens CH (1995). Implications for small reductions in diastolic blood pressure for primary prevention. Arch Intern Med.

[166] Cifkova R, Erdine S, Fagard R, Farsang C, Heagerty AM, Kiowski W (2003). Hypertension Guidelines Committee. Practice guidelines for primary care physicians: 2003 ESH/ESC hypertension guidelines. J Hypertens.

[167] WHO (2000). Obesity. Preventing and managing the global epidemic. Technical Report Series No. 894.

[168] Jensen MG, Ryan DH, Apovian CM, Ard JD, Comuzzie AG, Donato KA (2014). 2013 AHA/ACC/TOS guideline for the management of overweight and obesity in adults: a report of the American College of Cardiology/American Heart Association Task Force on Practice Guidelines and The Obesity Society. Circulation..

[169] Bacchi E, Cavendon V, Zancanaro C, Moghetti P, Milanese C. Comparison between dual-energy X-ray absorptiometry and skinfold thickness in assessing body fat in overweigh/obese adult patients with type-2 diabetes. Sci Rep. 2017;17424. 10.1038/s41598-017-17788-y. 10.1038/s41598-017-17788-y PMC5727309 29234125

[170] National Cholesterol Education Program (NCEP) Expert Panel on Detection, Evaluation, and Treatment of High Blood Cholesterol in Adults (Adult Treatment Panel III) (2002). Third report of the National Cholesterol Education Program (NCEP) Expert Panel on Detection, Evaluation, and Treatment of High Blood Cholesterol in Adults (Adult Treatment Panel III) final report. Circulation..

[171] Abbai NS, Nyirenda M, Reddy T, Ramjee G (2018). Good correlation between the Afinion AS100 analyser and the ABX Pentra 400 analyser for the measurement of glycosylated haemoglobin and lipid levels in older adults in Durban, South Africa. S Afr Med J.

[172] Foerster V, Severn M (2016). Point-of-care glycated hemoglobin testing to diagnose type 2 diabetes. CADTH Issues in Emerging Health Technologies.

[173] Rodondi N, Gencer B, Collet TH, Battegay E (2011). Ab welchem Cholesterinwert soll in der Schweiz eine Behandlung erfolgen?. Swiss Med Forum.

[174] Magno S, Ceccarini G, Pelosini C, Jaccheri R, Vitti J, Fierabracci P, et al. LDL-cholesterol lowering effect of a new dietary supplement: an open label, controlled, randomized, cross-over clinical trial in patients with mild-to-moderate hypercholesterolemia. Lipids Health Dis. 2018;17. 10.1186/s12944-018-0775-8. 10.1186/s12944-018-0775-8 PMC5968477 29793488

[175] Nathan DM (2015). Diabetes: Advances in diagnosis and treatment. JAMA..

[176] World Health Organization (2011). Use of glycated haemoglobin (HbA1c) in the diagnosis of diabetes mellitus.

[177] Little RR, Rohlfing CL, Sacks DB (2011). Status of hemoglobin A1c measurement and goals for improvement: from chaos to order for improving diabetes care. Clin Chem.

[178] Giese M, Unternahrer E, Huttig H, Beck J, Brand S, Calabrese P (2014). BDNF: an indicator of insomnia?. Mol Psychiatry.

[179] Giese Maria, Unternaehrer Eva, Brand Serge, Calabrese Pasquale, Holsboer-Trachsler Edith, Eckert Anne (2013). The Interplay of Stress and Sleep Impacts BDNF Level. PLoS ONE.

[180] LeRoith D, McGuinness M, Shemer J, Stannard B, Lanau F, Faria TN (1992). Insulin-like growth factors. Biol Signals.

[181] Karg K, Burmeister M, Shedden K, Sen S (2011). The serotonin transporter promoter variant (5-HTTLPR), stress, and depression meta-analysis revisited: evidence of genetic moderation. Arch Gen Psychiatry.

[182] Hsieh H-F, Shannon SE (2005). Three approaches to qualitative content analysis. Qual Health Res.

[183] Cohen J (1988). Statistical power analysis for the behavioral sciences.

[184] Gupta SK (2011). Intention-to-treat concept: a review. Perspect Clin Res.

[185] White I. R., Horton N. J., Carpenter J., statistics r. i. m. a. s., Pocock S. J. (2011). Strategy for intention to treat analysis in randomised trials with missing outcome data. BMJ.

